# A composition‐matching algorithm, MatchIDR, identifies prion‐like domains that localize to stress granules

**DOI:** 10.1002/pro.70567

**Published:** 2026-04-07

**Authors:** Sean M. Cascarina, Kacy R. Paul, Larissa L. Ford, Eric D. Ross

**Affiliations:** ^1^ Department of Biochemistry and Molecular Biology Colorado State University Fort Collins Colorado USA

**Keywords:** composition‐driven activity, intrinsically disordered region, liquid‐liquid phase separation, membraneless organelle, prion‐like domain, stress granule

## Abstract

Intrinsically disordered regions (IDRs) play important molecular roles in cells even though they do not adopt a stable structure. Relative to structured regions, IDRs have skewed amino acid compositions favoring polar and charged amino acids. This feature is a major contributor to the biophysical behavior and in vivo activity of IDRs, but the relationship between composition and activity depends strongly on which amino acids are enriched within the IDRs. Here, we present a new search algorithm, MatchIDR, that takes as input one or more IDR sequences and finds the nearest compositional matches within a proteome. Using MatchIDR with both artificially designed and native yeast IDRs as query sequences, we successfully identify IDRs from multiple organisms that localize (or do not localize) to yeast stress granules, as expected from the known activities of the query sequences. Our results demonstrate that composition‐based proteome searches can be an effective strategy for identifying new IDRs with similar in vivo activities. MatchIDR is available at https://github.com/RossLabCSU/MatchIDR.

## 1 INTRODUCTION

Intrinsically disordered regions (IDRs) are protein segments that do not adopt a stable tertiary structure in the absence of binding partners (Holehouse & Kragelund, 2024; Lemke et al., 2024; van der Lee et al., 2014). Although unified by the property of conformational heterogeneity, IDRs can vary substantially with respect to primary sequence, amino acid composition, conformational preferences, biophysical properties, and molecular functions (Holehouse & Kragelund, 2024). Many IDRs have “modular architectures” comprised of multiple subregions with distinct compositional biases, likely resulting in distinct behaviors and functions co‐existing within a single, contiguous IDR (McConnell & Parker, 2023). IDRs tend to evolve faster and tolerate more mutations compared to structured domains (Bellay et al., 2011; Brown et al., 2002, 2011; van der Lee et al., 2014). However, IDRs have important biological functions and typically still contain conserved features despite divergence in their primary sequences (Langstein‐Skora et al., 2026; Moesa et al., 2012; Zarin et al., 2019, 2021).

One important activity recently linked to IDRs is the formation of, or recruitment to, membraneless organelles (MLOs) such as stress granules (SGs), nucleoli, processing bodies, nuclear speckles, and many others (Banani et al., 2017). Many MLOs are thought to form via liquid–liquid phase separation (LLPS) of specific proteins or RNAs (Banani et al., 2017; Begovich & Wilhelm, 2020; Freibaum et al., 2021; Guillén‐Boixet et al., 2020; Sanders et al., 2020; Xing et al., 2020; Yang et al., 2020). These key nucleating proteins/RNAs are often referred to as “scaffolds,” whereas proteins and other molecules that are subsequently recruited to MLOs are referred to as “clients” (Banani et al., 2016, 2017; Ditlev et al., 2018; Xing et al., 2020). Current models suggest that IDRs engage in weak, multivalent interactions that can aid in the assembly of MLOs or the recruitment to pre‐formed MLOs (Bremer et al., 2022; Choi et al., 2020; Elbaum‐Garfinkle et al., 2015; Martin et al., 1979; Molliex et al., 2015; Nott et al., 2015; Pak et al., 2016; Protter et al., 2018). Importantly, these multivalent interactions are derived from the cumulative effect of key compositional features that are distributed across the length of the IDR. Consequently, for these types of domains, the order of amino acids (i.e., the primary sequence) may be less important than the overall amino acid composition (Cascarina & Ross, 2025).

As proof of principle, we recently screened a large selection of yeast prion‐like domains (PrLDs) for the ability to localize to SGs (Boncella et al., 2020). PrLDs are a specific subcategory of IDRs that have compositional similarity to yeast prion domains; PrLDs are typically enriched in Q and N residues, with secondary enrichment in other uncharged polar and aromatic amino acids (Alberti et al., 2009; Cascarina & Ross, 2014; Harrison & Gerstein, 2003; Michelitsch & Weissman, 2000). SGs are enriched in hundreds of different proteins and RNAs (Jain et al., 2016; Khong et al., 2017; Markmiller et al., 2018; Marmor‐Kollet et al., 2020; Protter & Parker, 2016). Multiple proteins that preferentially localize to SGs contain PrLDs (Harrison & Shorter, 2017; Li et al., 2013), and some (but not all) PrLDs are sufficient to drive the protein's localization to SGs (Boncella et al., 2020; Shattuck et al., 2019). Comparison of the PrLDs that localized to yeast SGs to the PrLDs that did not localize to SGs illuminated the compositional features of PrLDs that favor or disfavor SG localization. These compositional features were sufficient to develop a composition‐based algorithm to predict PrLD localization to SGs, as well as design completely artificial PrLDs with or without SG‐localization activity, with no consideration for primary sequence (Boncella et al., 2020). Furthermore, the degree of localization of PrLDs to SGs could be tuned in a stepwise fashion by gradual changes in hydrophobic content: a key feature of PrLDs contributing to SG recruitment (Baer et al., 2024).

Based on our previous successes in both elucidating and validating the compositional features driving PrLD recruitment to SGs, we reasoned that a composition‐based search might identify new IDRs/PrLDs with the ability to localize to SGs (Cascarina & Ross, 2025). Here we develop a new algorithm, MatchIDR, which scans whole proteomes, identifies, and ranks protein regions that share the highest compositional identity (CI) with a user‐defined query protein sequence. We show that this approach is effective at identifying sets of domains that do or do not localize to yeast SGs in accordance with the activities of the query proteins, regardless of the proteome from which the PrLD was identified. These results suggest that a composition‐matching strategy is capable of identifying IDRs with specific activities even when their primary sequences differ substantially.

## 2 RESULTS

### 2.1 The MatchIDR algorithm

**FIGURE 1:**
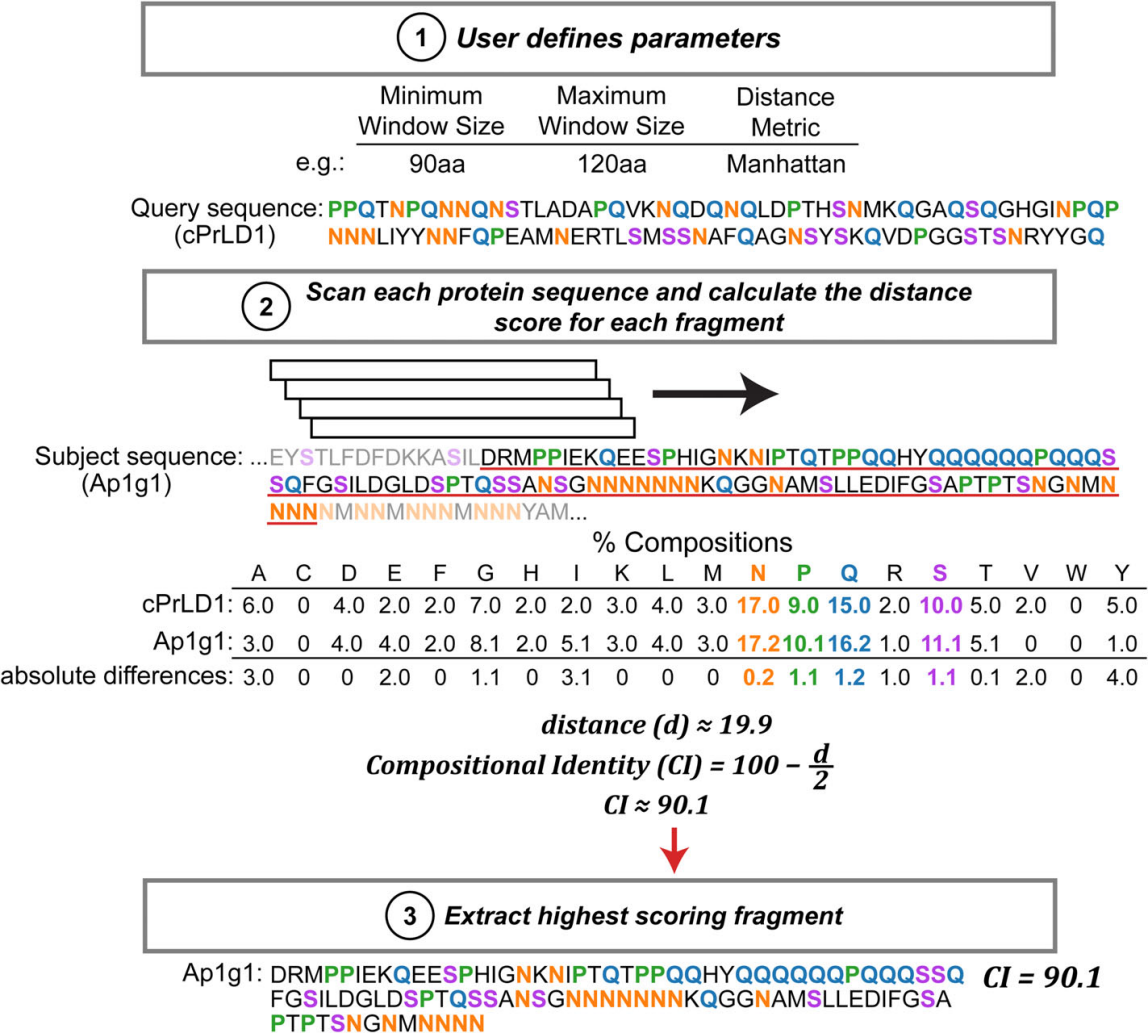
The MatchIDR algorithm. MatchIDR requires a query sequence and a proteome (or any FASTA‐formatted file) for composition‐matching searches. Additional parameters such as minimum window size, maximum window size, and distance metric can also be specified. MatchIDR scans each protein using the specified window size range and calculates the compositional identity compared to the query sequence. Prominent compositional features (exceeding 10% of the query or subject sequences) are colored for ease of comparison.

Many existing bioinformatic tools search for genes or proteins of interest based on sequence homology to a query sequence (Altschul et al., 1990; Buchfink et al., 2014; Eddy, 2011; Edgar, 2010; Kiełbasa et al., 2011; Li et al., 2024; Pearson & Lipman, 1988; Steinegger & Söding, 2017). To our knowledge, no general‐purpose tool has been designed to search for CI regardless of primary‐sequence identity and/or evolutionary relatedness. To address this need, we developed the MatchIDR algorithm, which can search whole proteomes and identify the region in each protein with the highest CI to an IDR of interest (Figure 1). CI is based on the total Manhattan distance between the percent compositions for the 20 canonical amino acids (see Section 4). Identified regions are then sorted from highest to lowest CI, revealing the regions of the proteome that are the closest compositional matches to the query sequence. A thorough discussion of MatchIDR parameters and output is included in Supporting Information S1: see “Supplementary Discussion” and Figures S1–S4.

### 2.2 MatchIDR identifies yeast PrLDs with SG‐localization activity

**FIGURE 2:**
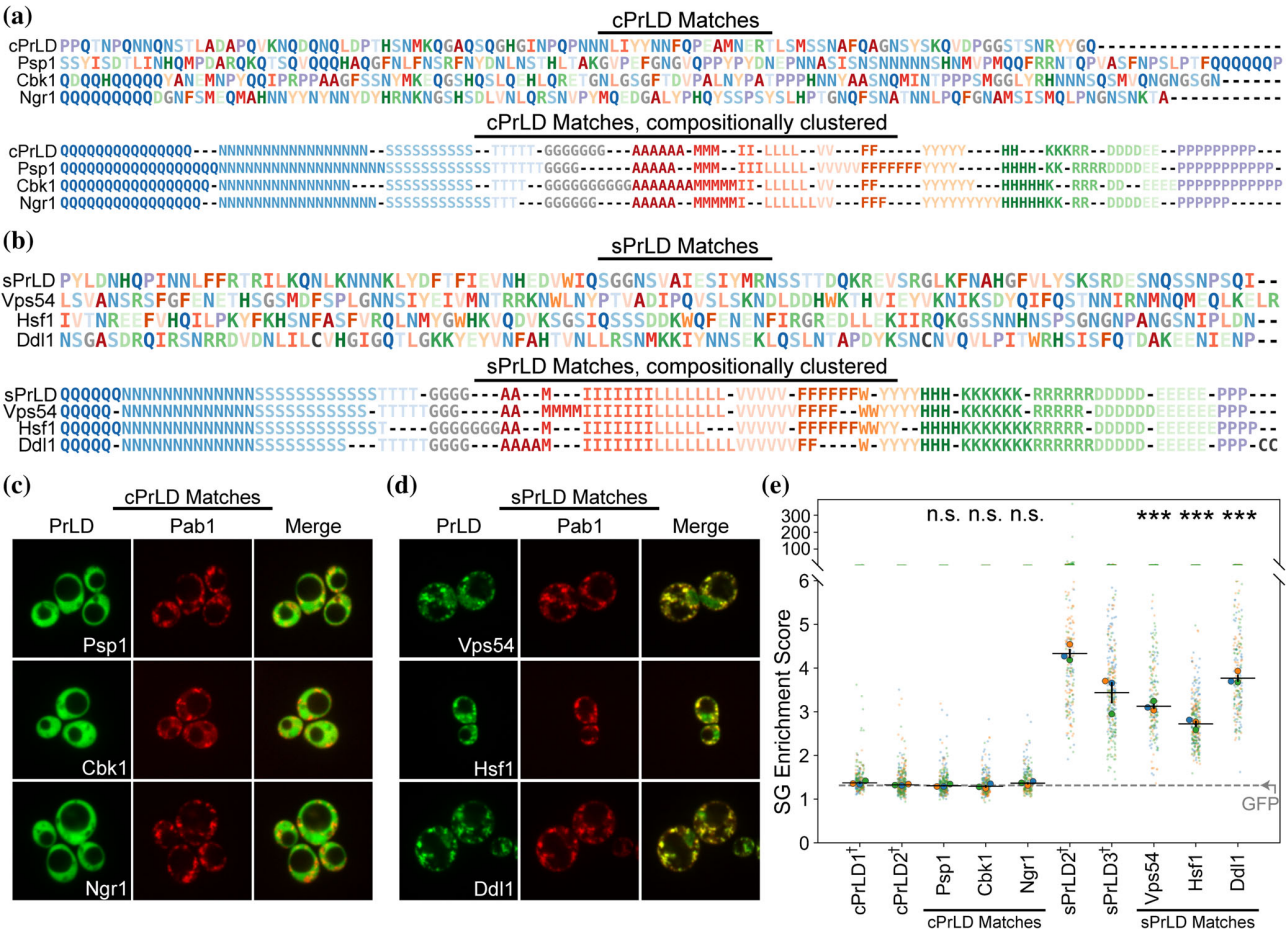
MatchIDR identifies yeast prion‐like domains (PrLDs) with stress granule (SG)‐localization activity that matches the synthetic PrLDs (sPrLD) or control PrLDs (cPrLD) query sequences. (a) Original sequences (*top*) and composition‐clustered sequence representations (*bottom*) of cPrLD and the corresponding top three MatchIDR matches from yeast. (b) Original sequences (*top*) and composition‐clustered sequence representations (*bottom*) of sPrLD and the corresponding top three MatchIDR matches from yeast. (c) Representative yeast microscopy images depicting protein localization of the three cPrLD matches and the SG marker, Pab1, after heat shock. (d) Protein localization of the three sPrLD matches after heat shock. (e) Quantification of SG enrichment scores for the cPrLD and sPrLD matches (see Section 4). The horizontal dotted line represents the average SG enrichment for green fluorescent protein (GFP) alone. All statistical comparisons are relative to cPrLD2. ^†^Data for cPrLD and sPrLD are derived from our previous publication and are shown here for comparison (Baer et al., 2024). *(p* ≥ 0.05, “n.s.”; *p* < 0.05, *; *p* < 0.01, **; *p* < 0.001, ***)

In prior studies, we elucidated compositional features of PrLDs that favor or disfavor localization to SGs (Baer et al., 2024; Boncella et al., 2020). We screened a library of PrLDs for the ability to localize to SGs during acute heat shock. We then designed 100‐amino acid synthetic PrLDs (sPrLDs) representing the average composition of yeast PrLDs from the screen that preferentially localized to SGs, and 100‐amino acid control PrLDs (cPrLDs) representing the average composition of yeast PrLDs that did not preferentially localize to SGs. As expected, the sPrLDs localize to SGs during acute heat shock, while the cPrLDs do not localize to SGs. The sPrLD sequences have more hydrophobic and charged amino acids along with fewer polar residues and prolines compared to the cPrLD sequences (Figure 2a,b).

Since compositional features are sufficient to explain the majority of SG‐localization activity for PrLDs, we reasoned that PrLDs with known SG‐localization activity could be used as query sequences to identify compositionally similar regions using MatchIDR. The artificial sPrLD and cPrLD sequences were used as query sequences to identify the top three matches in the yeast proteome for each query PrLD (Table S1; see Section 4 for search details). Coding sequences for the composition‐matched PrLDs were cloned into a plasmid that tagged them with green fluorescent protein (GFP), then transformed into a strain expressing a cytoplasmic marker (Rpl1b‐BFP) and an SG marker (Pab1‐mCherry) expressed from endogenous loci. These markers enable quantification of the degree of SG‐localization activity encoded by each PrLD, expressed as an “SG enrichment score” where higher values indicate greater enrichment in SGs (Baer et al., 2024).

The top matches for the sPrLD and cPrLD sequences exhibit no apparent primary sequence homology but strongly shared compositional features (Figure 2a,b). All three matches for the cPrLD query sequence remain diffuse after acute heat shock even though SGs still form in stressed cells (Figure 2c). This lack of SG localization results in low SG enrichment scores consistent with the original cPrLDs and GFP alone (Figure 2e and Table S2), neither of which are enriched in SGs to a detectable degree. In contrast, the three matches for the sPrLD query sequence form visible foci that colocalize with the Pab1 SG marker after heat shock (Figure 2d). Corresponding SG enrichment scores are consistent with clear SG‐localization activity but indicate slight differences in the degree of enrichment within SGs (Figure 2e). All MatchIDR matches remain completely diffuse or mostly diffuse in the absence of heat stress (Figure S5A,B): Ddl1 exhibited occasional pre‐stress foci that were not enriched in Pab1 and did not interfere with re‐localization to SGs upon heat shock. Therefore, a composition‐matching strategy using completely artificial PrLD sequences is sufficient to identify native yeast PrLDs with stress‐responsive SG‐localization activity as well as PrLDs lacking SG‐localization activity.

### 2.3 MatchIDR identifies PrLDs from diverse organisms that also exhibit SG‐localization activity in yeast

PrLD localization to SGs is dictated by the overall chemical properties of a PrLD sequence, which is determined largely by amino acid composition. The MatchIDR algorithm requires no organism‐specific assumptions: it is equally effective at identifying the best‐matching regions regardless of whether the query IDR is derived from the same organism, a different organism, or is entirely synthetic. Therefore, we used MatchIDR with the sPrLD and cPrLD query sequences to identify the top three matches for each sequence from the human and slime mold (*Dictyostelium discoideum*) proteomes using the same search parameters applied to the yeast proteome (Tables S3 and S4).

**FIGURE 3:**
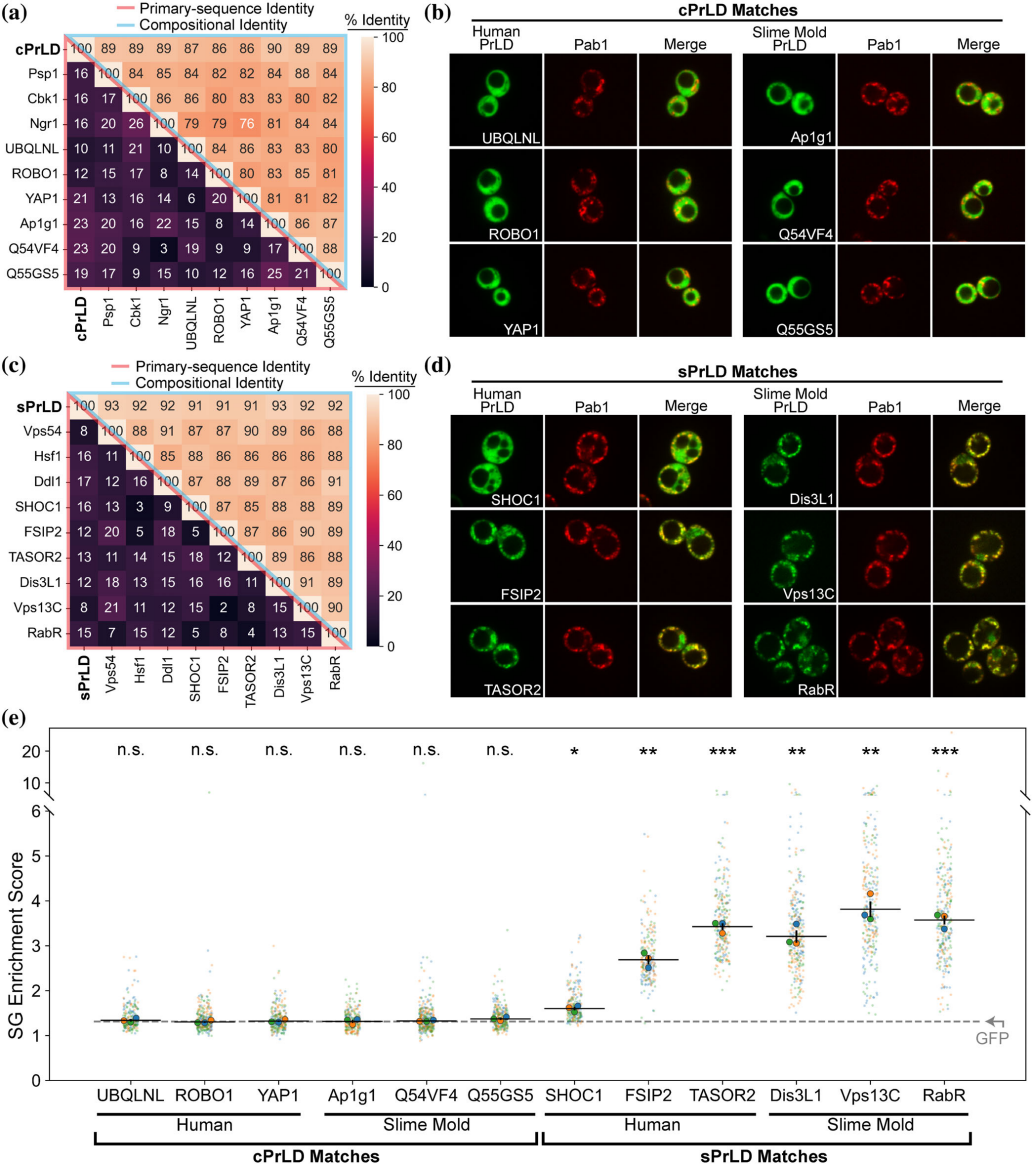
Stress granule (SG)‐localization activity of composition‐matched prion‐like domains (PrLDs) from eukaryotic organisms. (a) Heatmap depicting the primary‐sequence identity (*lower‐left half*) and the compositional identity (*upper‐right half*) for the top three PrLD matches for the control PrLDs (cPrLD) query sequence from yeast, humans, and slime mold. Matches consistently show high compositional identity but low primary‐sequence identity. (b) Protein localization of each cPrLD match from humans and slime mold with Pab1‐positive SGs that form in response to heat shock in yeast. (c) Heatmap depicting the primary‐sequence identity (*lower‐left half*) and the compositional identity (*upper‐right half*) for the top three PrLD matches for the sPrLD query sequence from yeast, humans, and slime mold. (d) Representative images displaying the colocalization of each sPrLD match from humans and slime mold with Pab1‐positive SGs after heat shock in yeast. (e) Quantification of SG enrichment scores for each PrLD. (*p* ≥ 0.05, “n.s.”; *p* < 0.05, *; *p* < 0.01, **; *p* < 0.001, ***)

All matches for the sPrLD or cPrLD sequences share strong CI with each other and with the original query sequence yet very low primary‐sequence identity in pairwise alignments (Figure 3a,c), indicating that the PrLDs identified by MatchIDR are not simply homologs with conserved primary sequences. Furthermore, the PrLD matches exhibit a degree of compositional diversity, with percent‐composition ranges for each amino acid typically centered at or near their corresponding query sequence (Figure S6). As observed for yeast, all six cPrLD matches from the human and slime mold proteomes remain diffuse during heat shock (Figure 3b) and exhibit low SG enrichment scores that are consistent with the original cPrLDs and with GFP alone (Figure 3e and Table S2), indicating that none of the cPrLD matches are enriched in SGs. In contrast, all six sPrLD matches from the two proteomes form foci during heat shock (Figure 3d) and have SG enrichment scores that are, on average, higher than scores for cPrLD and GFP alone (Figure 3e and Table S2). However, the degree of SG localization is not correlated with the magnitude of CI of each match compared to the corresponding query sequence (Figure S7). Most human and slime mold PrLDs were completely diffuse prior to heat shock (Figure S5A,B). While the slime mold matches with the sPrLD query protein formed non‐SG foci in a subset of cells prior to stress (Figure S5B), all three PrLDs robustly re‐localized to SGs after heat shock (Figure 3d). Collectively, these results suggest that the composition‐matching strategy is excellent at identifying candidates with or without the intended activity but does not precisely predict the level of SG‐localization activity, as deviations in composition could diminish, enhance, or have little effect on activity.

### 2.4 Native yeast PrLDs are effective query sequences for identifying human PrLDs with SG‐localization activity

In a prior study, we estimated SG propensity scores for each amino acid and used these scores to develop a composition‐based SG prediction algorithm for PrLDs, as well as design the sPrLD and cPrLD sequences (Boncella et al., 2020). These sPrLDs and cPrLDs were artificially engineered for high SG enrichment and no SG enrichment, respectively, making them ideal starting points to test the effectiveness of the composition‐matching strategy employed by MatchIDR. To examine whether this strategy would also work when native yeast PrLDs are used as query sequences, we chose three native yeast PrLDs with known SG‐localization activity and three native yeast PrLDs that do not localize to SGs (Boncella et al., 2020). Importantly, to determine if the composition‐matching strategy could be effective even in cases where the compositional features driving activity are not fully understood, we specifically chose PrLDs that exhibited SG‐enrichment activity that was poorly predicted by our SG prediction algorithm (Figure S8; Boncella et al., 2020). For each PrLD, a MatchIDR search was performed for the human proteome using the same parameters described for the sPrLD/cPrLD searches (Table S5). In each case, the top five human PrLD candidates were evaluated using our SG scoring algorithm (Boncella et al., 2020). The single candidate with an SG score most closely matching the SG score of the yeast query PrLD was tested for SG‐localization activity.

**FIGURE 4:**
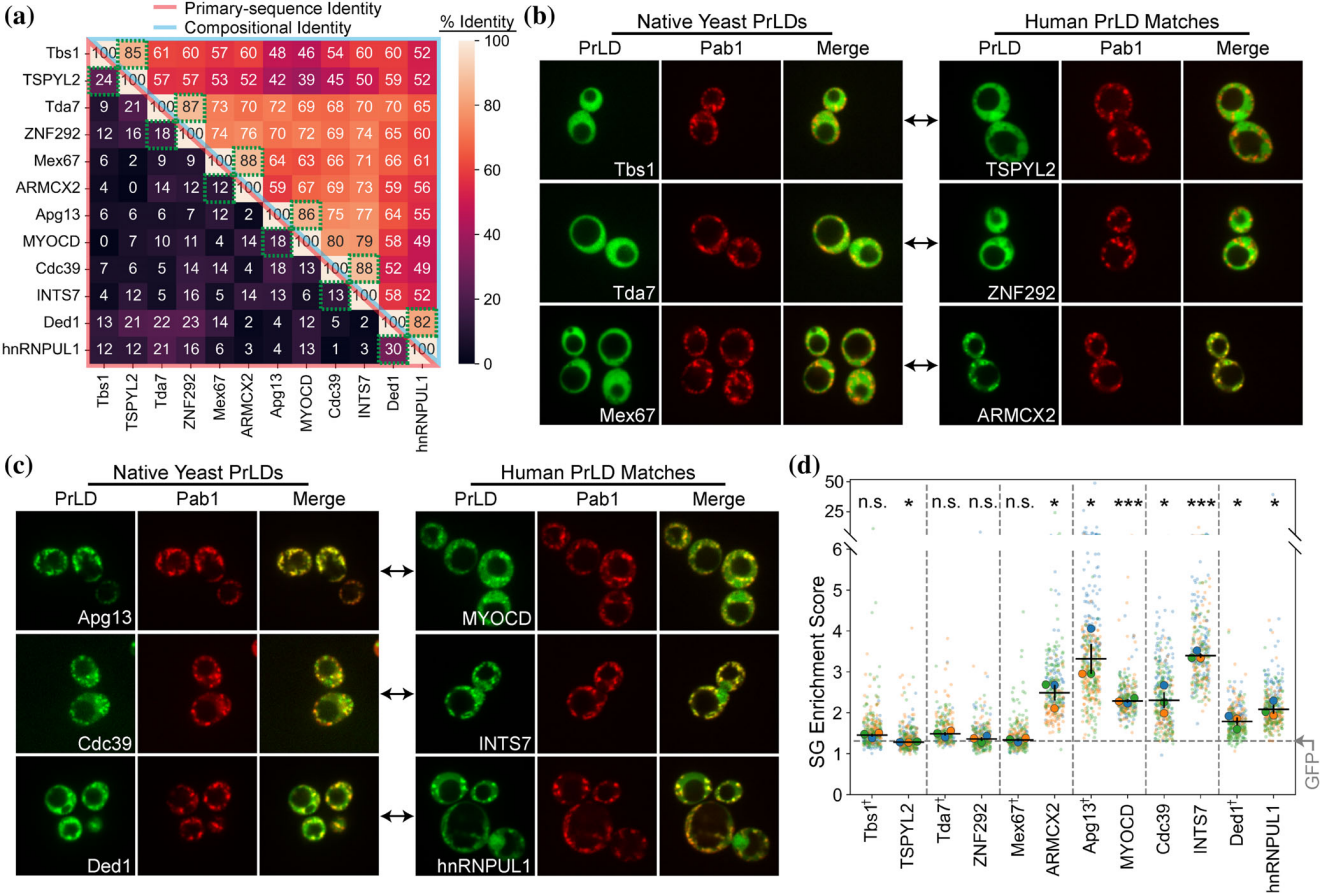
Stress granule (SG)‐localization activity of composition‐matched human prion‐like domains (PrLDs) for the native yeast query PrLDs. (a) Heatmap depicting the primary‐sequence identity (*lower‐left half*) and the compositional identity (*upper‐right half*) for the single best human PrLD matches for each yeast query sequence. Each yeast query PrLD appears before its paired human PrLD match, and these pairs are highlighted in adjacent, green, dotted‐line boxes. (b) Protein localization of the three native yeast PrLDs that do not localize to SGs (*left images*) and their corresponding top human PrLD matches (*right images*) after heat shock in yeast. SGs formed during heat shock in panels *b* and *c* are indicated by Pab1 foci. (c) Protein localization of the three native yeast PrLDs that localize to SGs (*left images*) and their corresponding top human PrLD matches (*right images*) after heat shock in yeast. (d) Quantification of SG enrichment scores for each PrLD. ^†^PrLDs with one replicate (blue) derived from our previous study (Baer et al., 2024). (*p* ≥ 0.05, “n.s.”; *p* < 0.05, *; *p* < 0.01, **; *p* < 0.001, ***)

As with the sPrLD and cPrLD searches, the single best match exhibited high CI but low primary‐sequence identity compared to the corresponding query sequence (Figure 4a). In contrast, comparisons across non‐similar PrLDs yielded lower compositional and primary‐sequence identities, reflecting the unique compositional profile of each native yeast PrLD used as query sequences. Among the set of human PrLDs expected to remain diffuse during heat shock, two out of the three human PrLD matches fail to form foci (Figure 4b) and have correspondingly low SG enrichment scores consistent with no detectable SG localization (Figure 4d and Table S2). Of the six human PrLDs, only ARMCX2 showed substantial foci formation prior to stress (Figure S5C,D), potentially contributing to its unexpected ability to localize to SGs. In contrast, all three human PrLD matches that were expected to localize to SGs form foci (Figure 4c) and have high SG enrichment scores (Figure 4d and Table S2), consistent with recruitment of these PrLDs to SGs. However, the degree of SG enrichment of the human PrLDs differed from that of the corresponding yeast query sequence, and differences in SG enrichment were not correlated with CI (Figure S9). Therefore, as with the sPrLD and cPrLD searches, MatchIDR run with native yeast PrLDs is effective at identifying composition‐matched regions with or without SG‐localization activity but does not predict the degree of SG enrichment. This highlights that MatchIDR can retrieve sequences with similar activity from whole‐proteome searches even in cases where the compositional requirements for the activity are poorly understood.

## 3 DISCUSSION

### 3.1 MatchIDR context and perspective

Protein sequences encoding well‐folded domains are often evolutionarily constrained and highly specific for the structures and activities that they encode. This tight relationship between amino acid sequence and protein activity limits the number and magnitude of sequence changes that can be tolerated by a sequence while still maintaining its activity. IDRs tend to be less constrained in this regard: for some IDRs, their amino acid composition is a stronger determinant of activity than their primary sequence (Cascarina & Ross, 2025).

Based on this hypothesis, we developed a computational tool, MatchIDR, to search for the nearest compositional match to a query sequence without regard for primary sequence. We show that MatchIDR is effective at identifying PrLDs with SG‐localization behavior similar to their corresponding query proteins. However, it is important to note that MatchIDR is not a prediction algorithm per se, nor is it limited to (or specific for) SG localization. CI is mathematically deterministic (i.e., not a function of probabilities or estimates), PrLD localization to SGs is predominantly a composition‐driven activity (Baer et al., 2024; Boncella et al., 2020), and other types of composition‐driven activities are known (Cascarina & Ross, 2025). The “success rate” of top‐ranking MatchIDR hits will largely be determined by how strongly the IDR activity depends on composition versus primary sequence. Furthermore, the activity of isolated IDRs is not always a good indicator of their activities in their native, full‐length proteins. For example, MatchIDR searches with the sPrLD query sequence identified three new yeast PrLDs that localized to SGs, but none of their native proteins are found in experimentally determined yeast SG datasets, presumably since none of these proteins are cytoplasmic (YeastRGB v1.3; Dubreuil et al., 2019): Hsf1 is nuclear and forms nuclear condensates in response to heat shock (Chowdhary et al., 2022; Rubio et al., 2024), Vps54 is in a Golgi‐associated complex, and Ddl1 is a mitochondrial matrix protein. Conversely, yeast SG proteins with PrLDs do not always rank highly in the same MatchIDR results (Figure S10), reflecting the fact that not all PrLDs drive SG localization and many other types of domains (especially RNA‐binding domains) can drive SG localization. We view this as a strength of MatchIDR: it was remarkably successful at identifying IDRs encoding a composition‐driven activity even when additional factors such as native protein localization or neighboring protein domains might mask that activity.

Although MatchIDR can perform cross‐organism searches, the goal of this search tool is not necessarily to identify homologous domains: rather, it is to identify domains with the greatest similarity in terms of compositional features. For query proteins or domains with composition‐driven activities, top‐scoring candidates are likely to encode similar activities through the features that they share with the query proteins. Multiple recent studies apply classification or clustering to large IDR datasets to infer IDR activities associated with specific clusters (Ruff et al., 2026; Singleton & Eisen, 2024; Zarin et al., 2019, 2021). Although these approaches have clear utility, they serve a fundamentally different purpose: MatchIDR is a proteome search algorithm designed to identify new IDRs with specific features, whereas classification/clustering approaches organize large datasets of pre‐defined IDRs into groups with similar features or activities. These tasks are complementary: by analogy, BLAST (Altschul et al., 1990; Camacho et al., 2009) searches identify similar primary sequences, whereas classification databases like Pfam (Paysan‐Lafosse et al., 2025; Sonnhammer et al., 1997) and SCOP (Chandonia et al., 2022; Murzin et al., 1995) add contextual information based on known examples. Like BLAST, MatchIDR requires no a priori knowledge about which features are relevant for the activity. We view MatchIDR as the compositional counterpart of BLAST, identifying matching regions based on CI rather than primary‐sequence identity. Both allow searches solely based on a sequence with a known activity, thereby allowing the user to generate a list of similar sequences for subsequent experimental or bioinformatic analysis.

Consistent with a composition‐driven model of PrLD localization to SGs, MatchIDR was remarkably successful at identifying PrLDs with SG‐localization activity that mirrors their query proteins. This approach was effective regardless of the organism that was searched and despite low primary‐sequence homologies among the matches, suggesting that the effect was driven largely by the chemical composition of the identified domains rather than conserved primary‐sequence motifs or yeast‐specific binding partners. However, it is important to note that SG‐localization activity may have different requirements in different organisms. For consistency, we tested all domains in our yeast model organism, where the original compositional features of SG‐localization activity were initially elucidated. While many human and slime mold PrLDs localized to SGs in yeast, these same PrLDs may not localize to SGs in their native host organisms. Organism‐specific differences in intracellular chemical environment, molecular composition, and regulatory factors likely influence the compositional features that drive a protein's activity in each organism. Therefore, validation of MatchIDR candidates is likely to be most successful when the experimental model system matches the source of the query proteins used in the MatchIDR searches (either domains native to that organism, or predictive compositional features derived from that organism).

### 3.2 Sequence features affecting PrLD localization to SGs

Mechanistically, how could PrLD localization to SGs be dictated by amino acid composition? SGs and other MLOs are thought to form via LLPS of specific molecular components. Multiple protein features and architectures have been proposed to explain the localization of IDRs to phase‐separated MLOs. For example, in the sticker‐and‐spacer model, key residues (“stickers”) drive weak, multivalent interactions with components of MLOs, while other residues in the domain act as “spacers” that promote intrinsic disorder (Bremer et al., 2022; Choi et al., 2020). Another model for MLO localization is the phase‐partitioning model, where proteins preferentially partition into one of the liquid phases based on the biophysical properties of the coexisting liquid compartments and the proteins themselves (Anderson et al., 2025; Mukherjee & Schäfer, 2023; Villegas & Levy, 2022). In contrast to simplified in vitro models of LLPS by purified components, PrLD localization to SGs in yeast does not seem to be concentration dependent (Baer et al., 2024), suggesting that SG localization of PrLDs is not driven by homotypic interactions promoting LLPS (i.e., they are not acting as key scaffolds nucleating SGs). Rather, the data suggest that PrLDs act as clients recruited to yeast SGs through phase partitioning and/or sticker‐and‐spacer interactions (which are not mutually exclusive). Both of these models are compatible with a composition‐driven model of PrLD recruitment to SGs (Baer et al., 2024).

While specific sequence patterns (e.g., charge patterning) are important for some IDR activities (Chong & Mir, 2021; Das & Pappu, 2013; Greig et al., 2020; Holehouse et al., 2021; Jankowski et al., 2024; Jonas et al., 2023; Lichtinger et al., 2021; Lotthammer et al., 2024; Lyons et al., 2023; Martin et al., 1979; Nott et al., 2015; Pak et al., 2016; Pesce et al., 2024; Ruff et al., 2026; Sherry et al., 2017; Tesei et al., 2024; von Bülow et al., 2025; Zheng et al., 2020), sequence patterning effects have not been rigorously characterized for PrLD localization to yeast SGs. Primary sequence clearly has some effect on the degree of SG enrichment by PrLDs: our two synthetic sPrLDs, which have identical compositions but different primary sequences, exhibit different levels of enrichment within SGs. Similarly, the MatchIDR matches sometimes exhibit different levels of SG enrichment despite having closely matching amino acid compositions. The PrLDs that localize to SGs exhibit only minor differences with respect to commonly analyzed sequence patterns compared to the PrLDs that do not localize to SG (Figure S11). The substantial overlap between these two groups suggests that composition alone explains the differences in SG localization better than any of these individual sequence patterns. Even for IDR activities with unknown or suspected dependence on sequence patterning, MatchIDR can be used to identify compositionally similar domains that span a range of patterning values. Testing multiple top‐ranking MatchIDR hits may aid in the elucidation of patterns important for a given IDR activity.

Collectively, our results provide further evidence that PrLD localization to SGs is driven by amino acid composition. However, many other protein activities have been associated with IDRs containing specific compositional profiles (Cascarina et al., 2021; Cascarina & Ross, 2018, 2024; Ruff et al., 2026; Zarin et al., 2019, 2021). For example, a proteome search strategy based solely on enrichment of arginine and serine identified new proteins with arginine/serine‐rich (RS) domains involved in RNA processing and nuclear speckle localization (Cascarina & Ross, 2022). A similar strategy searching for proteins with a high charge content and mixture of positively and negatively charged residues identified “mixed charge” domains also associated with nuclear speckle localization (Greig et al., 2020). Proteins known to localize to different types of condensates possess unique molecular grammars and molecular signatures (Cascarina & Ross, 2018; Lee et al., 2022; Ruff et al., 2026; Vashishtha & Sabari, 2025). Therefore, protein localization to other types of biomolecular condensates, as well as non‐condensate related activities (Cascarina & Ross, 2025), may be compositionally driven activities as well. When applied to these types of proteins and activities, MatchIDR is a powerful tool to uncover additional protein regions with shared compositional features and encoded activities.

## 4 MATERIALS AND METHODS

### 4.1 Yeast strains, cloning, and growth conditions

Experiments testing the localization of PrLDs to SGs were performed as previously described (Baer et al., 2024). Briefly, each PrLD identified by MatchIDR was cloned into a vector to express the PrLD with an N‐terminal GFP tag from the *ADH1* promoter. For the native yeast PrLDs used as query sequences for MatchIDR searches, we utilized previously constructed vectors expressing the PrLD with an N‐terminal GFP tag from the *SUP35* promoter (Boncella et al., 2020): in our recent study, the choice of promoter did not appreciably affect PrLD enrichment in SGs (Baer et al., 2024). These vectors were transformed into a strain expressing the cytoplasmic marker (Rpl1b‐BFP) and SG marker (Pab1‐mCherry) from their endogenous loci. Cells were grown overnight in SC‐Leu media (synthetic complete media lacking leucine) then diluted into fresh media, grown to mid‐log phase, and subjected to a 30‐min heat shock at 46°C immediately prior to imaging. At least three experimental replicates were performed for each PrLD, with a minimum of 30 quantifiable cells per replicate.

### 4.2 Quantification of SG enrichment scores

All microscopy images were collected on an Olympus (IX83) inverted spinning‐disk confocal microscope using a 100× objective. SG enrichment scores were calculated from microscopy images using our previously developed pipeline (Baer et al., 2024). Yeast cells were initially identified using YeastSpotter (Lu et al., 2019). Prior to quantification, cells were screened using a variety of criteria to ensure quality single‐cell data (see (Baer et al., 2024) for a full description), with one minor screening change: cells with little or no PrLD‐GFP expression were defined as those with a median cytoplasmic GFP intensity <1000 and a 90th percentile (rather than the median) GFP intensity in SGs <1000. The cytoplasm of each cell was defined as pixels with >0.8× the median intensity for all pixels in the cell for the Rpl1b‐BFP cytoplasmic marker. SGs were classified as regions with pixel intensities >2× the median intensity in the cell for the Pab1‐mCherry SG marker. SG enrichment for the PrLD‐GFP was calculated for each cell as the 90th percentile of GFP intensities in the SGs divided by the median GFP intensity in non‐SG cytoplasmic regions (Baer et al., 2024). SG enrichment scores are plotted as “SuperPlots” (Lord et al., 2020), where small colored dots represent the SG enrichment scores of individual cells (with different colors representing distinct experimental replicates), large colored dots represent the median of SG scores within a replicate, and small horizontal lines representing the mean of these medians for each construct. The horizontal dotted line represents the average of the median SG enrichment scores for GFP alone, as reported in (Baer et al., 2024). For all statistical comparisons, the median for each replicate was calculated from log_2_‐transformed, single‐cell SG enrichment scores. Medians were then averaged across replicates to yield a mean SG enrichment score. The mean SG enrichment score for each PrLD was compared to the mean SG enrichment score of cPrLD2 using a two‐sided Welch's *t*‐test. Statistical significance indicators in all figures summarize raw *p*‐values from these comparisons (Table S2).

### 4.3 Calculation of primary‐sequence identity and compositional identity

PrLDs were compared to the original query sequences and to each other in a pairwise fashion with respect to both primary sequence and composition. Primary‐sequence identity was extracted from pairwise alignments using the EMBOSS Needle program (Rice et al., 2000). CI was calculated according to the following equation:

$$\mathrm{Let}\;A=\begin{array}{l} \{\mathrm{Ala},\mathrm{Cys},\mathrm{Asp},\mathrm{Glu},\mathrm{Phe},\mathrm{Gly},\mathrm{His},\mathrm{Ile},\mathrm{Lys},\mathrm{Leu},\\ \mathrm{Met},\mathrm{Asn},\mathrm{Pro},\mathrm{Gln},\mathrm{Arg},\mathrm{Ser},\mathrm{Thr},\mathrm{Val},\mathrm{Trp},\mathrm{Tyr}\} \end{array}$$

$$\mathrm{CI}=100-\frac{\sum_{a\in A}\left|c_{a_{q}}-c_{a_{s}}\right|}{2}$$

where *A* represents the set of canonical amino acids and *c* represents the percent composition of amino acid *a* in the query *q* or subject *s* sequences. The sum term in the numerator represents the total Manhattan distance for the percent compositions for the 20 canonical amino acids in a subject sequence compared to the query sequence.

### 4.4 MatchIDR search parameters for sPrLD, cPrLD, and native yeast PrLD query sequences

The minimum length of PrLDs capable of driving localization to SGs has not been empirically determined. We used three main observations from our previous study (Boncella et al., 2020) to determine the window size range used for MatchIDR searches: (1) multiple native yeast PrLDs that are 81 amino acids in length can localize to SGs; (2) two sPrLDs that are 100 amino acids in length are strongly enriched in SGs; and (3) the median length of native yeast PrLDs that localize to SGs was ~122 amino acids. Therefore, we chose a narrow window size range of 90–120 amino acids (inclusive) for MatchIDR searches to identify candidate PrLDs to test in SG localization experiments. All MatchIDR searches were performed using the default Manhattan distance metric. Proteomes involved in searches include the yeast (*Saccharomyces cerevisiae*), slime mold (*Dictyostelium discoideum*), and human (*Homo sapiens*) proteomes (UniProt IDs UP000002311_559292, UP000002195_44689, and UP000005640_9606, respectively). The MatchIDR Python script is available at https://github.com/RossLabCSU/MatchIDR.

## CONFLICT OF INTEREST STATEMENT

The authors declare no conflicts of interest.

## Supporting information


**Figure S1.** Dependence of MatchIDR runtime on proteome size. Runtime of each MatchIDR search is plotted against proteome size in total amino acids (AAs) comprising the proteome. Each dot for a given organism represents a MatchIDR search with a different query protein. Ordinary least squares regression was used to determine a line of best fit.
**Figure S2.** Dependence of MatchIDR runtime on window size. Runtime of each MatchIDR search is plotted against window size. Each dot represents a MatchIDR search with a different query protein performed on the human proteome. Points were fitted with a second‐order polynomial to capture the non‐linear nature of the relationship.
**Figure S3.** Dependence of MatchIDR ranking on window size differences. For each window size (40, 60, 80, 100, 120, 140, 160, and 180), the absolute best MatchIDR hit was identified and searched for in MatchIDR results from window sizes ±80 around the given window size. The percentile of the original best hit was calculated from each of the searches using alternative window sizes. A window size difference of zero represents the original search and always has a rank percentile of 100.
**Figure S4.** Dependence of MatchIDR ranking on distance metric. (A) Mean rank of the best compositional match (Manhattan distance) among the MatchIDR results using the Euclidean distance, plotted as a function of window size. (B) Bar plot showing the difference in ranking between the top MatchIDR hits using Manhattan distance and their rankings according to Euclidean distance for the 27 PrLDs experimentally tested in this study. The ranks of top hits from each original MatchIDR search were subtracted from the ranks of those same proteins in the corresponding MatchIDR results using the Euclidean distance (“Rank Difference”).
**Figure S5.** Representative images of yeast expressing MatchIDR‐identified PrLDs at the standard 30°C growth temperature. Most PrLDs exhibited diffuse cytoplasmic localization at 30°C. For PrLDs with a small but reproducible subset of cells forming foci at 30°C (Ddl1, Dis3L1, Vps13C, RabR, and ARMCX2), representative images were selected to show at least one cell with a focus but do not reflect the frequency of foci‐forming cells among the population.
**Figure S6.** Amino acid composition of sPrLD matches and cPrLD matches. Percent composition for each amino acid was calculated for MatchIDR matches from yeast, humans, and slime mold and grouped based on the original query protein (sPrLD matches in blue, cPrLD matches in red). For comparison, the composition values for sPrLD and cPrLD are represented as black stars.
**Figure S7.** SG enrichment score versus compositional identity for sPrLD and cPrLD matches. MatchIDR matches for sPrLD (blue) and cPrLD (red) from yeast, humans, and slime mold are plotted with respect to mean SG enrichment score and compositional identity relative to their corresponding reference sequences.
**Figure S8.** SG‐propensity scores for native yeast PrLDs with previously characterized SG‐localization activity. Predicted SG propensities for a set of native yeast PrLDs with SG enrichment (“SG‐positive PrLDs”) or no detectable SG enrichment (“SG‐negative PrLDs”) were reported in our prior study (Boncella et al., 2020). The contribution of each amino acid to SG localization was estimated based on their enrichment or depletion in the SG‐positive PrLD sequences relative to the SG‐negative PrLD sequences. Predicted SG‐propensity scores are the average estimated contribution of each residue across the PrLD sequence. The six native yeast PrLDs used as query sequences in MatchIDR searches (labeled proteins; this study) represent the most extreme inconsistencies between predicted SG propensity and observed SG‐localization activity.
**Figure S9.** SG enrichment score versus compositional identity for human PrLDs identified as top matches using native yeast PrLD query sequences. Each human MatchIDR match was compared to its corresponding yeast PrLD query sequence with respect to both SG enrichment score and compositional identity. Values on the *y*‐axis represent the difference in mean SG enrichment score for the human PrLD and the mean SG enrichment score for its yeast PrLD counterpart.
**Figure S10.** Ranking of yeast SG proteins containing predicted PrLDs among MatchIDR results for the sPrLD query protein. Yeast SG proteins from two different SG datasets (Wallace et al., 2015; Jain et al., 2016) were analyzed using two different prion prediction algorithms, PAPA and PLAAC (Toombs et al., 2010; Lancaster et al., 2014). Of the SG proteins with a predicted PrLD, the rank of each protein was determined among the MatchIDR results generated by using the sPrLD query protein, a window size range of 90–120, and the Manhattan distance metric.
**Figure S11.** Measures of sequence patterning among SG‐localizing and non‐SG‐localizing PrLDs. (A) Linear dispersion of charged (D/E/H/K/R), aromatic (F/W/Y), and hydrophobic (I/L/M/V) amino acids according to the linear dispersion method used in LCD‐Composer (Cascarina et al., 2021). (B) Patterning features *κ* (distribution of D/E relative to K/R), Ω_arom_ (distribution of F/W/Y relative to all other residues), and Ω_hyd_ (distribution of I/L/V relative to all other residues) according to localCIDER (Holehouse et al., 2017). Despite normalization, some values can exceed 1.0 when the residues involved in the patterning feature constitute a small fraction of the sequence (Ginell and Holehouse, 2020; Cohan et al., 2022). (C) Patterning of various groups of residues according to NARDINI+ (Cohan et al., 2022; Ruff et al., 2026). Residue groups are pol (S/T/N/Q/C/H), hyd (I/L/M/V), pos (K/R), neg (D/E), aro (F/W/Y). Patterning features with the same label repeated (e.g., pol‐pol) represent patterning of those residues relative to all other types of residues not in that group. NARDINI+ assigns an arbitrary *z*‐score of 0 to a sequence if the total fraction of residues belonging to either group in the patterning feature constitute <10% of the sequence composition. While all 36 patterning features were evaluated for the SG proteins, only those with at least one non‐zero value for the SG‐positive and SG‐negative groups are shown.

**Table S1.** MatchIDR search results from *Saccharomyces cerevisiae* for the sPrLD and cPrLD query proteins. See “MatchIDR Output” section on page 2 for a description of data fields.

**Table S2.** *p*‐Values for SG enrichment scores. Two‐tailed Welch's *t*‐tests were performed to determine if the degree of SG localization differed significantly from a protein with no detectable SG enrichment (cPrLD2). For each comparison, the SG enrichment scores of that protein are compared to the SG enrichment scores for cPrLD2 from Figure 2.

**Table S3.** MatchIDR search results from *Homo sapiens* for the sPrLD and cPrLD query proteins. See “MatchIDR Output” section on page 2 for a description of data fields.

**Table S4.** MatchIDR search results from *Dictyostelium discoideum* for the sPrLD and cPrLD query proteins. See “MatchIDR Output” section on page 2 for a description of data fields.

**Table S5.** MatchIDR search results from *Homo sapiens* for the native yeast PrLD query proteins. See “MatchIDR Output” section on page 2 for a description of data fields.

## Data Availability

The data that support the findings of this study are available from the corresponding author upon reasonable request.

## References

[pro70567-bib-0001] Alberti S , Halfmann R , King O , Kapila A , Lindquist S . A systematic survey identifies prions and illuminates sequence features of prionogenic proteins. Cell. 2009;137:146–158. 10.1016/j.cell.2009.02.044 19345193 PMC2683788

[pro70567-bib-0002] Altschul SF , Gish W , Miller W , Myers EW , Lipman DJ . Basic local alignment search tool. J Mol Biol. 1990;215:403–410. 10.1016/S0022-2836(05)80360-2 2231712

[pro70567-bib-0003] Anderson S , Harrison M , Dignon GL . Hydration free energy is an incomplete predictor of globular protein incorporation into condensates. Biophys J. 2025;125:1–10. 10.1016/j.bpj.2025.12.005 41351266 PMC12798646

[pro70567-bib-0004] Baer MH , Cascarina SM , Paul KR , Ross ED . Rational tuning of the concentration‐independent enrichment of prion‐like domains in stress granules. J Mol Biol. 2024;436:168703. 10.1016/j.jmb.2024.168703 39004265 PMC11486480

[pro70567-bib-0005] Banani SF , Lee HO , Hyman AA , Rosen MK . Biomolecular condensates: organizers of cellular biochemistry. Nat Rev Mol Cell Biol. 2017;18:285–298. 10.1038/nrm.2017.7 28225081 PMC7434221

[pro70567-bib-0006] Banani SF , Rice AM , Peeples WB , Lin Y , Jain S , Parker R , et al. Compositional control of phase‐separated cellular bodies. Cell. 2016;166:651–663. 10.1016/j.cell.2016.06.010 27374333 PMC4967043

[pro70567-bib-0007] Begovich K , Wilhelm JE . An in vitro assembly system identifies roles for RNA nucleation and ATP in yeast stress granule formation. Mol Cell. 2020;79:991–1007. 10.1016/j.molcel.2020.07.017 32780990

[pro70567-bib-0008] Bellay J , Han S , Michaut M , Kim T , Costanzo M , Andrews BJ , et al. Bringing order to protein disorder through comparative genomics and genetic interactions. Genome Biol. 2011;12:R14. 10.1186/gb-2011-12-2-r14 21324131 PMC3188796

[pro70567-bib-0009] Boncella AE , Shattuck JE , Cascarina SM , Paul KR , Baer MH , Fomicheva A , et al. Composition‐based prediction and rational manipulation of prion‐like domain recruitment to stress granules. Proc Natl Acad Sci U S A. 2020;117:5826–5835. 10.1073/pnas.1912723117 32127480 PMC7084078

[pro70567-bib-0010] Bremer A , Farag M , Borcherds WM , Peran I , Martin EW , Pappu RV , et al. Deciphering how naturally occurring sequence features impact the phase behaviours of disordered prion‐like domains. Nat Chem. 2022;14:196–207. 10.1038/s41557-021-00840-w 34931046 PMC8818026

[pro70567-bib-0011] Brown CJ , Johnson AK , Dunker AK , Daughdrill GW . Evolution and disorder. Curr Opin Struct Biol. 2011;21:441–446. 10.1016/j.sbi.2011.02.005 21482101 PMC3112239

[pro70567-bib-0012] Brown CJ , Takayama S , Campen AM , Vise P , Marshall TW , Oldfield CJ , et al. Evolutionary rate heterogeneity in proteins with long disordered regions. J Mol Evol. 2002;55:104–110. 10.1007/s00239-001-2309-6 12165847

[pro70567-bib-0013] Buchfink B , Xie C , Huson DH . Fast and sensitive protein alignment using DIAMOND. Nat Methods. 2014;12:59–60. 10.1038/nmeth.3176 25402007

[pro70567-bib-0014] Camacho C , Coulouris G , Avagyan V , Ma N , Papadopoulos J , Bealer K , et al. BLAST+: architecture and applications. BMC Bioinformatics. 2009;10:421. 10.1186/1471-2105-10-421 20003500 PMC2803857

[pro70567-bib-0015] Cascarina SM , King DC , Osborne Nishimura E , Ross ED . LCD‐Composer: an intuitive, composition‐centric method enabling the identification and detailed functional mapping of low‐complexity domains. NAR Genom Bioinform. 2021;3:lqab048. 10.1093/nargab/lqab048 34056598 PMC8153834

[pro70567-bib-0016] Cascarina SM , Ross ED . Yeast prions and human prion‐like proteins: sequence features and prediction methods. Cell Mol Life Sci. 2014;71:2047–2063. 10.1007/s00018-013-1543-6 24390581 PMC4024371

[pro70567-bib-0017] Cascarina SM , Ross ED . Proteome‐scale relationships between local amino acid composition and protein fates and functions. PLoS Comput Biol. 2018;14:e1006256. 10.1371/journal.pcbi.1006256 30248088 PMC6171957

[pro70567-bib-0018] Cascarina SM , Ross ED . Expansion and functional analysis of the SR‐related protein family across the domains of life. RNA. 2022;28:1298–1314. 10.1261/rna.079170.122 35863866 PMC9479744

[pro70567-bib-0019] Cascarina SM , Ross ED . Identification of low‐complexity domains by compositional signatures reveals class‐specific frequencies and functions across the domains of life. PLoS Comput Biol. 2024;20:e1011372. 10.1371/journal.pcbi.1011372 38748749 PMC11132505

[pro70567-bib-0020] Cascarina SM , Ross ED . Protein activities driven by amino acid composition. J Biol Chem. 2025;301:110640. 10.1016/j.jbc.2025.110640 40885394 PMC12509984

[pro70567-bib-0021] Chandonia J‐M , Guan L , Lin S , Yu C , Fox NK , Brenner SE . SCOPe: improvements to the structural classification of proteins – extended database to facilitate variant interpretation and machine learning. Nucleic Acids Res. 2022;50:D553–D559. 10.1093/nar/gkab1054 34850923 PMC8728185

[pro70567-bib-0022] Choi J‐M , Holehouse AS , Pappu RV . Physical principles underlying the complex biology of intracellular phase transitions. Annu Rev Biophys. 2020;49:107–133. 10.1146/annurev-biophys-121219-081629 32004090 PMC10715172

[pro70567-bib-0023] Chong S , Mir M . Towards decoding the sequence‐based grammar governing the functions of intrinsically disordered protein regions. J Mol Biol. 2021;433:166724. 10.1016/j.jmb.2020.11.023 33248138

[pro70567-bib-0024] Chowdhary S , Kainth AS , Paracha S , Gross DS , Pincus D . Inducible transcriptional condensates drive 3D genome reorganization in the heat shock response. Mol Cell. 2022;82:P4386–4399.E7. 10.1016/j.molcel.2022.10.013 PMC9701134 36327976

[pro70567-bib-0025] Das RK , Pappu RV . Conformations of intrinsically disordered proteins are influenced by linear sequence distributions of oppositely charged residues. Proc Natl Acad Sci U S A. 2013;110:13392–13397. 10.1073/pnas.1304749110 23901099 PMC3746876

[pro70567-bib-0026] Ditlev JA , Case LB , Rosen MK . Who's in and who's out—compositional control of biomolecular condensates. J Mol Biol. 2018;430:4666–4684. 10.1016/j.jmb.2018.08.003 30099028 PMC6204295

[pro70567-bib-0027] Dubreuil B , Sass E , Nadav Y , Heidenreich M , Georgeson JM , Weill U , et al. YeastRGB: comparing the abundance and localization of yeast proteins across cells and libraries. Nucleic Acids Res. 2019;47:D1245–D1249. 10.1093/nar/gky941 30357397 PMC6324022

[pro70567-bib-0028] Eddy SR . Accelerated profile HMM searches. PLoS Comput Biol. 2011;7:e1002195. 10.1371/journal.pcbi.1002195 22039361 PMC3197634

[pro70567-bib-0029] Edgar RC . Search and clustering orders of magnitude faster than BLAST. Bioinformatics. 2010;26:2460–2461. 10.1093/bioinformatics/btq461 20709691

[pro70567-bib-0030] Elbaum‐Garfinkle S , Kim Y , Szczepaniak K , Chen CC‐H , Eckmann CR , Myong S , et al. The disordered P granule protein LAF‐1 drives phase separation into droplets with tunable viscosity and dynamics. Proc Natl Acad Sci U S A. 2015;112:7189–7194. 10.1073/pnas.1504822112 26015579 PMC4466716

[pro70567-bib-0031] Freibaum BD , Messing J , Yang P , Kim HJ , Taylor JP . High‐fidelity reconstitution of stress granules and nucleoli in mammalian cellular lysate. J Cell Biol. 2021;220:e202009079. 10.1083/jcb.202009079 33502444 PMC7845923

[pro70567-bib-0032] Greig JA , Nguyen TA , Lee M , Holehouse AS , Posey AE , Pappu RV , et al. Arginine‐enriched mixed‐charge domains provide cohesion for nuclear speckle condensation. Mol Cell. 2020;77:1237–1250.e4. 10.1016/j.molcel.2020.01.025 32048997 PMC10715173

[pro70567-bib-0033] Guillén‐Boixet J , Kopach A , Holehouse AS , Wittmann S , Jahnel M , Schlüßler R , et al. RNA‐induced conformational switching and clustering of G3BP drive stress granule assembly by condensation. Cell. 2020;181:346–361. 10.1016/j.cell.2020.03.049 32302572 PMC7181197

[pro70567-bib-0034] Harrison PM , Gerstein M . A method to assess compositional bias in biological sequences and its application to prion‐like glutamine/asparagine‐rich domains in eukaryotic proteomes. Genome Biol. 2003;4:R40. 10.1186/gb-2003-4-6-r40 12801414 PMC193619

[pro70567-bib-0035] Harrison AF , Shorter J . RNA‐binding proteins with prion‐like domains in health and disease. Biochem J. 2017;474:1417–1438. 10.1042/BCJ20160499 28389532 PMC5639257

[pro70567-bib-0036] Holehouse AS , Ginell GM , Griffith D , Böke E . Clustering of aromatic residues in prion‐like domains can tune the formation, state, and organization of biomolecular condensates. Biochemistry. 2021;60:3566–3581. 10.1021/acs.biochem.1c00465 34784177 PMC8638251

[pro70567-bib-0037] Holehouse AS , Kragelund BB . The molecular basis for cellular function of intrinsically disordered protein regions. Nat Rev Mol Cell Biol. 2024;25:187–211. 10.1038/s41580-023-00673-0 37957331 PMC11459374

[pro70567-bib-0038] Jain S , Wheeler JR , Walters RW , Agrawal A , Barsic A , Parker R . ATPase‐modulated stress granules contain a diverse proteome and substructure. Cell. 2016;164:487–498. 10.1016/j.cell.2015.12.038 26777405 PMC4733397

[pro70567-bib-0039] Jankowski MS , Griffith D , Shastry DG , Pelham JF , Ginell GM , Thomas J , et al. Disordered clock protein interactions and charge blocks turn an hourglass into a persistent circadian oscillator. Nat Commun. 2024;15:3523‐. 10.1038/s41467-024-47761-z 38664421 PMC11045787

[pro70567-bib-0040] Jonas F , Carmi M , Krupkin B , Steinberger J , Brodsky S , Jana T , et al. The molecular grammar of protein disorder guiding genome‐binding locations. Nucleic Acids Res. 2023;51:4831–4844. 10.1093/nar/gkad184 36938874 PMC10250222

[pro70567-bib-0041] Khong A , Matheny T , Jain S , Mitchell SF , Wheeler JR , Parker R . The stress granule transcriptome reveals principles of mRNA accumulation in stress granules. Mol Cell. 2017;68:808–820. 10.1016/j.molcel.2017.10.015 29129640 PMC5728175

[pro70567-bib-0042] Kiełbasa SM , Wan R , Sato K , Horton P , Frith MC . Adaptive seeds tame genomic sequence comparison. Genome Res. 2011;21:487–493. 10.1101/gr.113985.110 21209072 PMC3044862

[pro70567-bib-0043] Langstein‐Skora I , Schmid A , Huth F , Shabani D , Spechtenhauser L , Likhodeeva M , et al. Sequence and chemical specificity define the functional landscape of intrinsically disordered regions. Nat Cell Biol. 2026;28:323–337. 10.1038/s41556-025-01867-8 41688823 PMC12904797

[pro70567-bib-0044] Lee B , Jaberi‐Lashkari N , Calo E . A unified view of low complexity regions (LCRs) across species. Elife. 2022;11:e77058. 10.7554/eLife.77058 36098382 PMC9470157

[pro70567-bib-0045] Lemke EA , Babu MM , Kriwacki RW , Mittag T , Pappu RV , Wright PE , et al. Intrinsic disorder: a term to define the specific physicochemical characteristic of protein conformational heterogeneity. Mol Cell. 2024;84:1188–1190. 10.1016/j.molcel.2024.02.024 38579677

[pro70567-bib-0046] Li YR , King OD , Shorter J , Gitler AD . Stress granules as crucibles of ALS pathogenesis. J Cell Biol. 2013;201:361–372. 10.1083/jcb.201302044 23629963 PMC3639398

[pro70567-bib-0047] Li J , Wang Z , Fan X , Yao R , Zhang G , Fan R , et al. Rapid multiple protein sequence search by parallel and heterogeneous computation. Bioinformatics. 2024;40:40. 10.1093/bioinformatics/btae151 PMC11021808 38547405

[pro70567-bib-0048] Lichtinger SM , Garaizar A , Collepardo‐Guevara R , Reinhardt A . Targeted modulation of protein liquid–liquid phase separation by evolution of amino‐acid sequence. PLoS Comput Biol. 2021;17:e1009328. 10.1371/journal.pcbi.1009328 34428231 PMC8415608

[pro70567-bib-0049] Lord SJ , Velle KB , Dyche Mullins R , Fritz‐Laylin LK . SuperPlots: communicating reproducibility and variability in cell biology. J Cell Biol. 2020;219:e202001064. 10.1083/JCB.202001064/151717 32346721 PMC7265319

[pro70567-bib-0050] Lotthammer JM , Ginell GM , Griffith D , Emenecker RJ , Holehouse AS . Direct prediction of intrinsically disordered protein conformational properties from sequence. Nat Methods. 2024;21:465–476. 10.1038/s41592-023-02159-5 38297184 PMC10927563

[pro70567-bib-0051] Lu AX , Zarin T , Hsu IS , Moses AM . YeastSpotter: accurate and parameter‐free web segmentation for microscopy images of yeast cells. Bioinformatics. 2019;35:4525–4527. 10.1093/bioinformatics/btz402 31095270 PMC6821424

[pro70567-bib-0052] Lyons H , Veettil RT , Pradhan P , Fornero C , De La Cruz N , Ito K , et al. Functional partitioning of transcriptional regulators by patterned charge blocks. Cell. 2023;186:327–345. 10.1016/j.cell.2022.12.013 36603581 PMC9910284

[pro70567-bib-0053] Markmiller S , Soltanieh S , Server KL , Mak R , Jin W , Fang MY , et al. Context‐dependent and disease‐specific diversity in protein interactions within stress granules. Cell. 2018;172:590–604.e13. 10.1016/j.cell.2017.12.032 29373831 PMC5969999

[pro70567-bib-0054] Marmor‐Kollet H , Siany A , Kedersha N , Knafo N , Rivkin N , Danino YM , et al. Spatiotemporal proteomic analysis of stress granule disassembly using APEX reveals regulation by SUMOylation and links to ALS pathogenesis. Mol Cell. 2020;80:876–891.e6. 10.1016/j.molcel.2020.10.032 33217318 PMC7816607

[pro70567-bib-0055] Martin EW , Holehouse AS , Peran I , Farag M , Incicco JJ , Bremer A , et al. Valence and patterning of aromatic residues determine the phase behavior of prion‐like domains. Science. 1979;367:694–699. 10.1126/science.aaw8653 PMC7297187 32029630

[pro70567-bib-0056] McConnell BS , Parker MW . Protein intrinsically disordered regions have a non‐random, modular architecture. Bioinformatics. 2023;39:btad732. 10.1093/bioinformatics/btad732 38039154 PMC10719218

[pro70567-bib-0057] Michelitsch MD , Weissman JS . A census of glutamine/asparagine‐rich regions: implications for their conserved function and the prediction of novel prions. Proc Natl Acad Sci U S A. 2000;97:11910–11915. 10.1073/pnas.97.22.11910 11050225 PMC17268

[pro70567-bib-0058] Moesa HA , Wakabayashi S , Nakai K , Patil A . Chemical composition is maintained in poorly conserved intrinsically disordered regions and suggests a means for their classification. Mol Biosyst. 2012;8:3262–3273. 10.1039/c2mb25202c 23076520

[pro70567-bib-0059] Molliex A , Temirov J , Lee J , Coughlin M , Kanagaraj AP , Kim HJ , et al. Phase separation by low complexity domains promotes stress granule assembly and drives pathological fibrillization. Cell. 2015;163:123–133. 10.1016/j.cell.2015.09.015 26406374 PMC5149108

[pro70567-bib-0060] Mukherjee S , Schäfer LV . Thermodynamic forces from protein and water govern condensate formation of an intrinsically disordered protein domain. Nat Commun. 2023;14:5892. 10.1038/s41467-023-41586-y 37735186 PMC10514047

[pro70567-bib-0061] Murzin AG , Brenner SE , Hubbard T , Chothia C . SCOP: a structural classification of proteins database for the investigation of sequences and structures. J Mol Biol. 1995;247:536–540. 10.1016/S0022-2836(05)80134-2 7723011

[pro70567-bib-0062] Nott TJ , Petsalaki E , Farber P , Jervis D , Fussner E , Plochowietz A , et al. Phase transition of a disordered Nuage protein generates environmentally responsive membraneless organelles. Mol Cell. 2015;57:936–947. 10.1016/j.molcel.2015.01.013 25747659 PMC4352761

[pro70567-bib-0063] Pak CW , Kosno M , Holehouse AS , Padrick SB , Mittal A , Ali R , et al. Sequence determinants of intracellular phase separation by complex coacervation of a disordered protein. Mol Cell. 2016;63:72–85. 10.1016/j.molcel.2016.05.042 27392146 PMC4973464

[pro70567-bib-0064] Paysan‐Lafosse T , Andreeva A , Blum M , Chuguransky SR , Grego T , Pinto BL , et al. The Pfam protein families database: embracing AI/ML. Nucleic Acids Res. 2025;53:D523–D534. 10.1093/nar/gkae997 39540428 PMC11701544

[pro70567-bib-0065] Pearson WR , Lipman DJ . Improved tools for biological sequence comparison. Proc Natl Acad Sci U S A. 1988;85:2444–2448. 10.1073/pnas.85.8.2444 3162770 PMC280013

[pro70567-bib-0066] Pesce F , Bremer A , Tesei G , Hopkins JB , Grace CR , Mittag T , et al. Design of intrinsically disordered protein variants with diverse structural properties. Sci Adv. 2024;10:9926. 10.1126/sciadv.adm9926 PMC11352843 39196930

[pro70567-bib-0067] Protter DSW , Parker R . Principles and properties of stress granules. Trends Cell Biol. 2016;26:668–679. 10.1016/j.tcb.2016.05.004 27289443 PMC4993645

[pro70567-bib-0068] Protter DSW , Rao BS , Van Treeck B , Lin Y , Mizoue L , Rosen MK , et al. Intrinsically disordered regions can contribute promiscuous interactions to RNP granule assembly. Cell Rep. 2018;22:1401–1412. 10.1016/j.celrep.2018.01.036 29425497 PMC5824733

[pro70567-bib-0069] Rice P , Longden I , Bleasby A . EMBOSS: the European Molecular Biology Open Software Suite. Trends Genet. 2000;16:276–277. 10.1016/S0168-9525(00)02024-2 10827456

[pro70567-bib-0070] Rubio LS , Mohajan S , Gross DS . Heat Shock Factor 1 forms nuclear condensates and restructures the yeast genome before activating target genes. Elife. 2024;12:RP92464. 10.7554/eLife.92464.4 39405097 PMC11479590

[pro70567-bib-0071] Ruff KM , King MR , Ying AW , Liu V , Pant A , Lieberman WE , et al. Molecular grammars of predicted intrinsically disordered regions that span the human proteome. Cell. 2026;189:323–342.e17. 10.1016/j.cell.2025.10.019 41232529 PMC13296966

[pro70567-bib-0072] Sanders DW , Kedersha N , Lee DSW , Strom AR , Drake V , Riback JA , et al. Competing protein‐RNA interaction networks control multiphase intracellular organization. Cell. 2020;181:306–324. 10.1016/j.cell.2020.03.050 32302570 PMC7816278

[pro70567-bib-0073] Shattuck JE , Paul KR , Cascarina SM , Ross ED . The prion‐like protein kinase Sky1 is required for efficient stress granule disassembly. Nat Commun. 2019;10:3614. 10.1038/s41467-019-11550-w 31399582 PMC6688984

[pro70567-bib-0074] Sherry KP , Das RK , Pappu RV , Barrick D . Control of transcriptional activity by design of charge patterning in the intrinsically disordered RAM region of the Notch receptor. Proc Natl Acad Sci U S A. 2017;114:E9243–E9252. 10.1073/pnas.1706083114 29078291 PMC5676888

[pro70567-bib-0075] Singleton MD , Eisen MB . Evolutionary analyses of intrinsically disordered regions reveal widespread signals of conservation. PLoS Comput Biol. 2024;20:e1012028. 10.1371/journal.pcbi.1012028 38662765 PMC11075841

[pro70567-bib-0076] Sonnhammer ELL , Eddy SR , Durbin R . Pfam: a comprehensive database of protein domain families based on seed alignments. Proteins: Struct Funct Genet. 1997;28:405–420. 10.1002/(sici)1097-0134(199707)28:3<405::aid-prot10>3.0.co;2-l 9223186

[pro70567-bib-0077] Steinegger M , Söding J . MMseqs2 enables sensitive protein sequence searching for the analysis of massive data sets. Nat Biotechnol. 2017;35:1026–1028. 10.1038/nbt.3988 29035372

[pro70567-bib-0078] Tesei G , Trolle AI , Jonsson N , Betz J , Knudsen FE , Pesce F , et al. Conformational ensembles of the human intrinsically disordered proteome. Nature. 2024;626:897–904. 10.1038/s41586-023-07004-5 38297118

[pro70567-bib-0079] van der Lee R , Buljan M , Lang B , Weatheritt RJ , Daughdrill GW , Dunker AK , et al. Classification of intrinsically disordered regions and proteins. Chem Rev. 2014;114:6589–6631. 10.1021/cr400525m 24773235 PMC4095912

[pro70567-bib-0080] Vashishtha S , Sabari BR . Disordered regions of condensate‐promoting proteins have distinct molecular signatures associated with cellular function. J Mol Biol. 2025;437:168953. 10.1016/j.jmb.2025.168953 39826710 PMC12232917

[pro70567-bib-0081] Villegas JA , Levy ED . A unified statistical potential reveals that amino acid stickiness governs nonspecific recruitment of client proteins into condensates. Protein Sci. 2022;31:e4361. 10.1002/pro.4361 35762716 PMC9207749

[pro70567-bib-0082] von Bülow S , Tesei G , Zaidi FK , Mittag T , Lindorff‐Larsen K . Prediction of phase‐separation propensities of disordered proteins from sequence. Proc Natl Acad Sci U S A. 2025;122:e2417920122. 10.1073/pnas.2417920122 40131954 PMC12002312

[pro70567-bib-0083] Xing W , Muhlrad D , Parker R , Rosen MK . A quantitative inventory of yeast P body proteins reveals principles of composition and specificity. Elife. 2020;9:e56525. 10.7554/eLife.56525 32553117 PMC7373430

[pro70567-bib-0084] Yang P , Mathieu C , Kolaitis RM , Zhang P , Messing J , Yurtsever U , et al. G3BP1 is a tunable switch that triggers phase separation to assemble stress granules. Cell. 2020;181:325–345. 10.1016/j.cell.2020.03.046 32302571 PMC7448383

[pro70567-bib-0085] Zarin T , Strome B , Nguyen Ba AN , Alberti S , Forman‐Kay JD , Moses AM . Proteome‐wide signatures of function in highly diverged intrinsically disordered regions. Elife. 2019;8:e46883. 10.7554/eLife.46883 31264965 PMC6634968

[pro70567-bib-0086] Zarin T , Strome B , Peng G , Pritišanac I , Forman‐Kay JD , Moses AM . Identifying molecular features that are associated with biological function of intrinsically disordered protein regions. Elife. 2021;10:e60220. 10.7554/eLife.60220 33616531 PMC7932695

[pro70567-bib-0087] Zheng W , Dignon G , Brown M , Kim YC , Mittal J . Hydropathy patterning complements charge patterning to describe conformational preferences of disordered proteins. J Phys Chem Lett. 2020;11:3408–3415. 10.1021/acs.jpclett.0c00288 32227994 PMC7450210

