# Supplementary material for: A composition‐matching algorithm, MatchIDR, identifies prion‐like domains that localize to stress granules
Source: Protein Sci. 2026 Apr 7;35(5):e70567. doi: 10.1002/pro.70567 (PMC13055198; doi:10.1002/pro.70567)
Supplement: Supplementary file 1 — Figure S1. Dependence of MatchIDR runtime on proteome size. Runtime of each MatchIDR search is plotted against proteome size in total amino acids (AAs) comprising the proteome. Each dot for a given organism represents a MatchIDR search with a different query protein. Ordinary least squares regression was used to determine a line of best fit. Figure S2. Dependence of MatchIDR runtime on window size. Runtime of each MatchIDR search is plotted against window size. Each dot represents a MatchIDR search with a different query protein performed on the human proteome. Points were fitted with a second‐order polynomial to capture the non‐linear nature of the relationship. Figure S3. Dependence of MatchIDR ranking on window size differences. For each window size (40, 60, 80, 100, 120, 140, 160, and 180), the absolute best MatchIDR hit was identified and searched for in MatchIDR results from window sizes ±80 around the given window size. The percentile of the original best hit was calculated from each of the searches using alternative window sizes. A window size difference of zero represents the original search and always has a rank percentile of 100. Figure S4. Dependence of MatchIDR ranking on distance metric. (A) Mean rank of the best compositional match (Manhattan distance) among the MatchIDR results using the Euclidean distance, plotted as a function of window size. (B) Bar plot showing the difference in ranking between the top MatchIDR hits using Manhattan distance and their rankings according to Euclidean distance for the 27 PrLDs experimentally tested in this study. The ranks of top hits from each original MatchIDR search were subtracted from the ranks of those same proteins in the corresponding MatchIDR results using the Euclidean distance (“Rank Difference”). Figure S5. Representative images of yeast expressing MatchIDR‐identified PrLDs at the standard 30°C growth temperature. Most PrLDs exhibited diffuse cytoplasmic localization at 30°C. For PrLDs with a small but r [file PRO-35-e70567-s006.docx]

**Supplementary Material**

**Table of Contents**

[Supplementary Discussion, Figs S1-S4 2](#_Toc220339438)

[*MatchIDR Output* 2](#_Toc220339439)

[*MatchIDR Computational Performance and Scaling* 3](#_Toc220339440)

[*Window Size Selection* 4](#_Toc220339441)

[*Distance Metrics* 5](#_Toc220339442)

[Fig S5 8](#_Toc220339443)

[Fig S6 9](#_Toc220339444)

[Fig S7 10](#_Toc220339445)

[Fig S8 11](#_Toc220339446)

[Fig S9 12](#_Toc220339447)

[Fig S10 13](#_Toc220339448)

[Fig S11 14](#_Toc220339449)

[Supplementary Table Legends 15](#_Toc220339450)

[References 16](#_Toc220339451)

Supplementary Discussion, Figs S1-S4

*MatchIDR Output*

MatchIDR produces a single, tab-separated values (.tsv) file for each search. The first eight lines start with “>” and document the runtime parameters, including:

| **Parameter Title** | **Description** |
| --- | --- |
| >*RUNTIME PARAMETERS* | Title marking the runtime parameters section of the results file. |
| >Job ID | A random numerical ID internally assigned to the search at runtime. |
| >Query IDR Sequence(s) | The amino acid sequence(s) of the IDR or IDRs used as queries. |
| >FASTA ID(s) of Query IDR(s) | FASTA headers for each of the query sequences. |
| >FASTA File | The FASTA file that was searched for matches (typically a proteome). |
| >Minimum Window Size | The smallest window size used in the search. |
| >Maximum Window Size | The largest window size used in the search. |
| >Distance Metric | The distance metric used to compare the composition vectors of two sequences. |

An empty line separates that runtime parameters from the column headers and subsequent MatchIDR data. Column headers include:

| **Column** | **Description** |
| --- | --- |
| Protein Description | The full FASTA header of the protein analyzed from the searched FASTA file. |
| UniProtID | The UniProtID of the searched protein. Only applicable when a UniProt proteome is searched, otherwise all values will be “N/A”. |
| Query Protein ID | The ID of the query protein that was used to identify the Best Fragment Match in the protein analyzed. This becomes relevant when more than one query protein is contained in the FASTA file specified as the query file at runtime (see description below). |
| Best Fragment Match | The sequence of the protein fragment with the highest compositional identity from the protein analyzed. |
| Compositional Identity | The compositional identity of the Best Fragment Match compared to the query protein sequence. When the Manhattan distance metric is used, compositional identity ranges from 0-100 and can be compared to primary-sequence identity. When the Euclidean distance metric is used, compositional identity exists on a different scale and is not comparable to primary-sequence identity, but rank ordering of compositional matches is still valid. |
| Distance Score for Best Fragment | The distance between the composition vectors for the query sequence and the Best Fragment Match. This distance is used to calculate Compositional Identity. Range will depend on the Distance Metric chosen at runtime. |
| Domain Boundaries for Best Fragment | The start and end positions of the Best Fragment Match in the protein analyzed. |
| Columns 8-29 | Single-letter abbreviation for the 20 canonical amino acids + O (pyrrolysine) and U (selenocysteine). Each column will contain the contribution of that amino acid to the Distance Score (absolute value of the difference between the query and subject sequences for Manhattan distance, squared difference between the two sequences for Euclidean distance). |

Query sequences are passed to MatchIDR by providing a FASTA file name. The FASTA file may contain more than one query protein: in these cases, each protein in the searched proteome will be evaluated independently for every query protein, and each result will have a separate line in the MatchIDR output file (one for each combination of query protein and protein analyzed from the searched proteome). Proteins with total lengths below the minimum window size are excluded from analysis and the MatchIDR output file.

*MatchIDR Computational Performance and Scaling*

To estimate the dependence of MatchIDR runtime on proteome size and window size, additional MatchIDR searches were performed on 12 eukaryotic proteomes with a variety of query proteins and parameters. Specifically, all PrLDs used as query sequences in MatchIDR searches in this study (sPrLD, cPrLD, Tbs1, Tda7, Mex67, Apg13, Cdc39, and Ded1) were used as queries in each search. Searches were performed on a basic desktop computer (Intel Core i7 8-core processor and 16GB RAM running Windows 11 Enterprise).

Dependence of MatchIDR runtime on proteome size was tested using a fixed 100-amino acid window size with all combinations of PrLD query sequences and eukaryotic proteomes. Average runtime increased linearly with proteome size (Fig S1). Although different query proteins were used in the MatchIDR searches, query protein characteristics have a negligible effect on runtime since all query proteins are represented as identically shaped composition vectors for calculations.


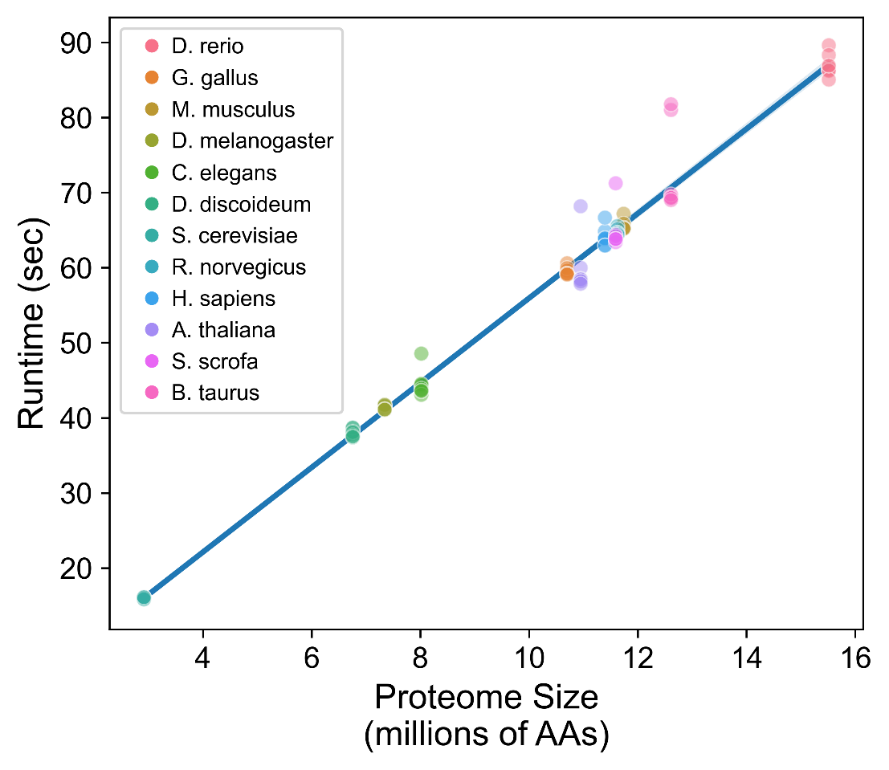


**Fig S1. Dependence of MatchIDR runtime on proteome size.** Runtime of each MatchIDR search is plotted against proteome size in total amino acids (AAs) comprising the proteome. Each dot for a given organism represents a MatchIDR search with a different query protein. Ordinary least squares regression was used to determine a line of best fit.

Dependence of MatchIDR runtime on window size was tested using all PrLD query proteins in the human proteome only and window sizes ranging from 20 to 200 (in increments of 20). Average runtime increases with window size, but in a non-linear fashion (Fig S2) for two main reasons. First, MatchIDR only evaluates proteins that are as large as the minimum window size. At larger window sizes, more proteins tend to be excluded from analysis (though this will depend on the protein size distribution of each proteome). Second, only sequence fragments that occupy a complete window are evaluated, resulting in fewer windows analyzed per protein.


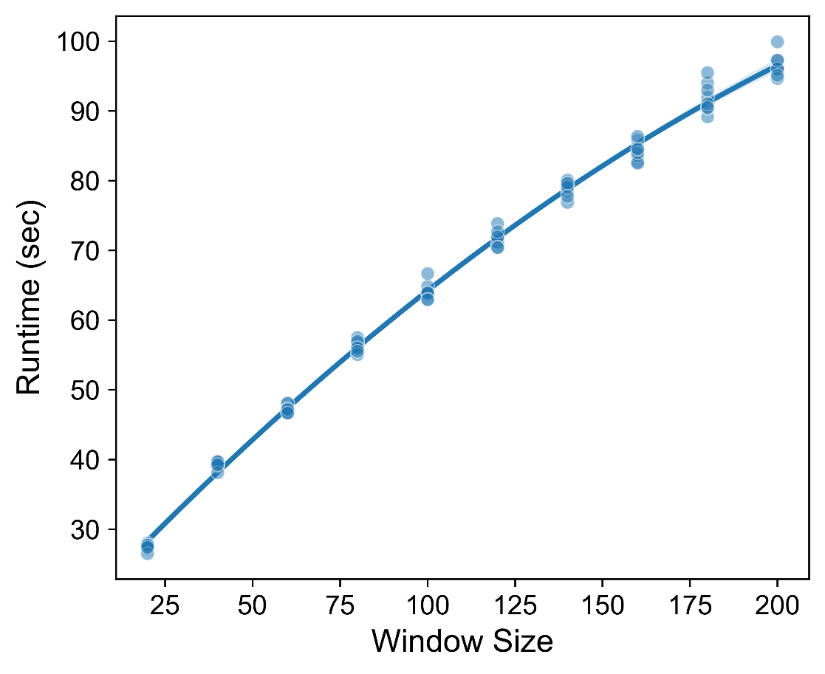


**Fig S2. Dependence of MatchIDR runtime on window size.** Runtime of each MatchIDR search is plotted against window size. Each dot represents a MatchIDR search with a different query protein performed on the human proteome. Points were fitted with a second-order polynomial to capture the non-linear nature of the relationship.

*Window Size Selection*

The distance metrics underpinning MatchIDR are equally valid and accurate for all positive window sizes. Window size ranges should be selected based primarily on the biological phenomenon being studied and the lengths of known sequences with a given protein activity. However, window size selection will determine the best compositional matches and their rankings when performing MatchIDR searches.

To determine how sensitive the rank order of MatchIDR hits is to window size, MatchIDR searches were performed on the yeast proteome for the eight query PrLDs using all possible window sizes from 20 to 200 amino acids. For each query PrLD, the top-ranking protein was identified for all window sizes in the range 20 to 200 in increments of 20. The percentile (with respect to rank) of this top-ranking protein was then determined in all search results from window sizes within 80 of the original window size. For example, a search with the Apg13 query PrLD and a 100-amino acid window size identified the top-ranking MatchIDR hit. The rank percentile of this protein was then calculated from the search results for all window sizes from 20 amino acids to 180 amino acids. This process is repeated for all query proteins and relevant window sizes.

Fig S3 shows the results from each query protein, as well as average percentiles across all query proteins. MatchIDR rankings drift the greatest when smaller window sizes are used or when results from large window sizes are compared to those from small window sizes. While most of the queries exhibit relatively stable rankings (particularly for large window sizes, rank volatility differs between query proteins suggesting that query protein features may influence ranking stability. For these reasons, we recommend using a range of window sizes (rather than a single window size) that is based on known or predicted protein lengths capable of encoding the specific protein activity being studied.

It should be emphasized that rank stability is not a reflection of MatchIDR algorithm performance: rather, different sequences are identified as the best compositional matches when using different window sizes. Regardless of the window size used, MatchIDR will always generate a mathematically accurate ranking of domains according to compositional identity (as defined by the chosen distance metric).


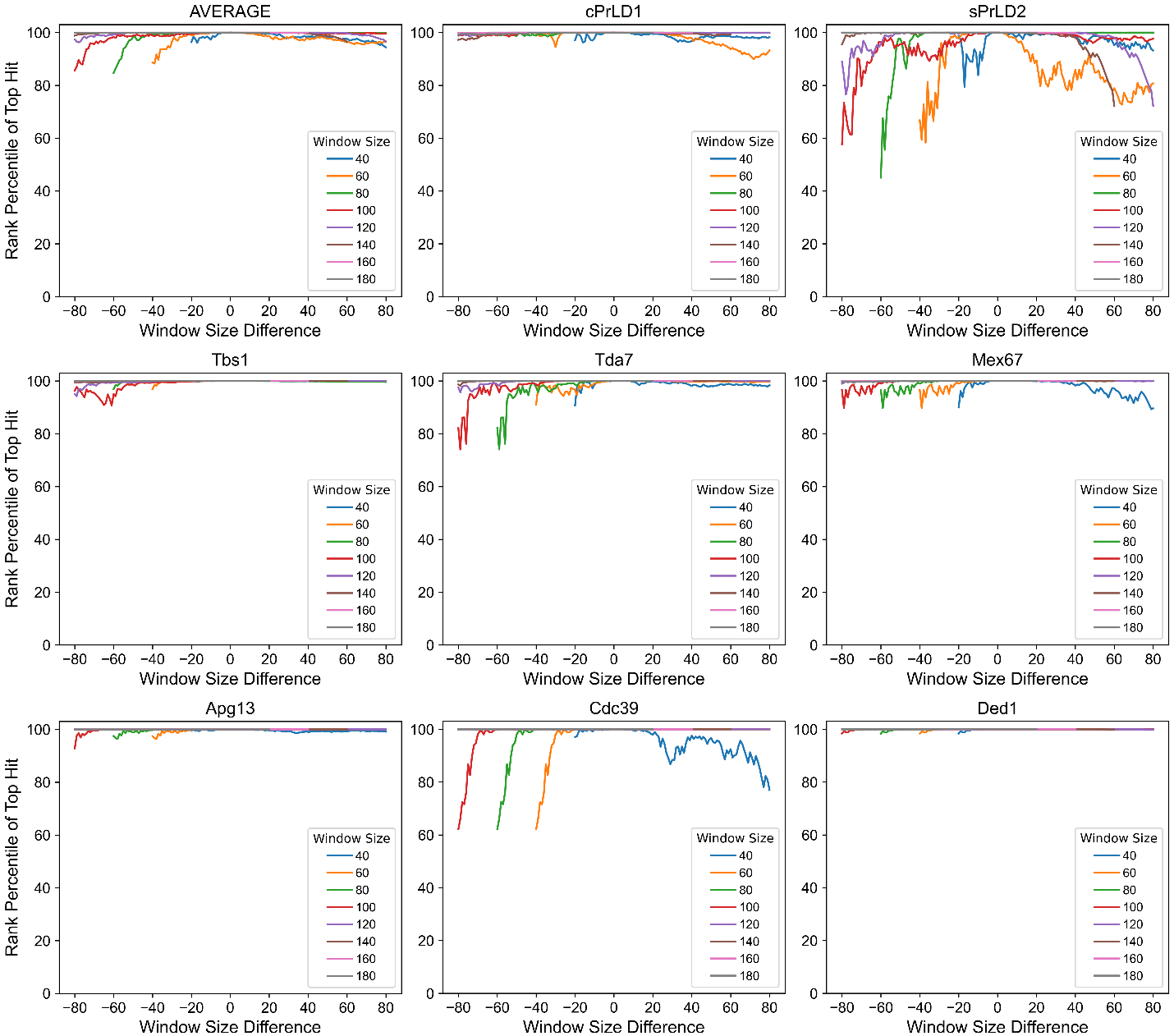


**Fig S3. Dependence of MatchIDR ranking on window size differences.** For each window size (40, 60, 80, 100, 120, 140, 160, and 180), the absolute best MatchIDR hit was identified and searched for in MatchIDR results from window sizes ±80 around the given window size. The percentile of the original best hit was calculated from each of the searches using alternative window sizes. A window size difference of zero represents the original search and always has a rank percentile of 100.

*Distance Metrics*

By default, MatchIDR uses the Manhattan distance to compare sequences and calculate compositional identity. In this context, the Manhattan distance has two main advantages compared to alternative distance metrics. First, the calculation of compositional identity from the Manhattan distance is on the same scale and directly comparable to primary sequence identity. Any change that affects both the sequence and composition to the same degree will result in equal decreases in primary-sequence identity and compositional identity (Cascarina and Ross, 2025). This correspondence is not maintained when using the Euclidean distance metric. Second, the Manhattan distance equally weights protein features regardless of their abundance in the sequences being compared. In contrast, the Euclidean distance is heavily influenced by the amino acids with the largest compositional differences between a pair of sequences. For some IDRs, this would effectively weight the most abundant feature(s) in the sequences even if that feature is not the most important determinant of protein activity.

Nevertheless, MatchIDR offers the Euclidean distance as an alternative option. To examine the effects of chosen distance metric on MatchIDR ranking, we performed two sets of MatchIDR searches.

First, all MatchIDR searches for PrLDs that were experimentally tested in this study were repeated with identical parameters but using the Euclidean distance metric. Specifically, the eight query PrLDs were used to search the yeast, human, and slime mold proteomes with a window size range of 90-120. The top three hits were evaluated for the sPrLD and cPrLD searches, whereas the absolute top hit was evaluated for the remaining query PrLDs. In a full two-thirds of searches (16 out of 24), the Euclidean distance retrieved the same top hits (and in the same rank order) from the proteome as the Manhattan distance (Fig S4A). An additional five searches had the best Manhattan distance hits among the top three Euclidean distance hits. Therefore, the choice of distance metric did not have a major effect on which PrLDs were experimentally examined in this study.


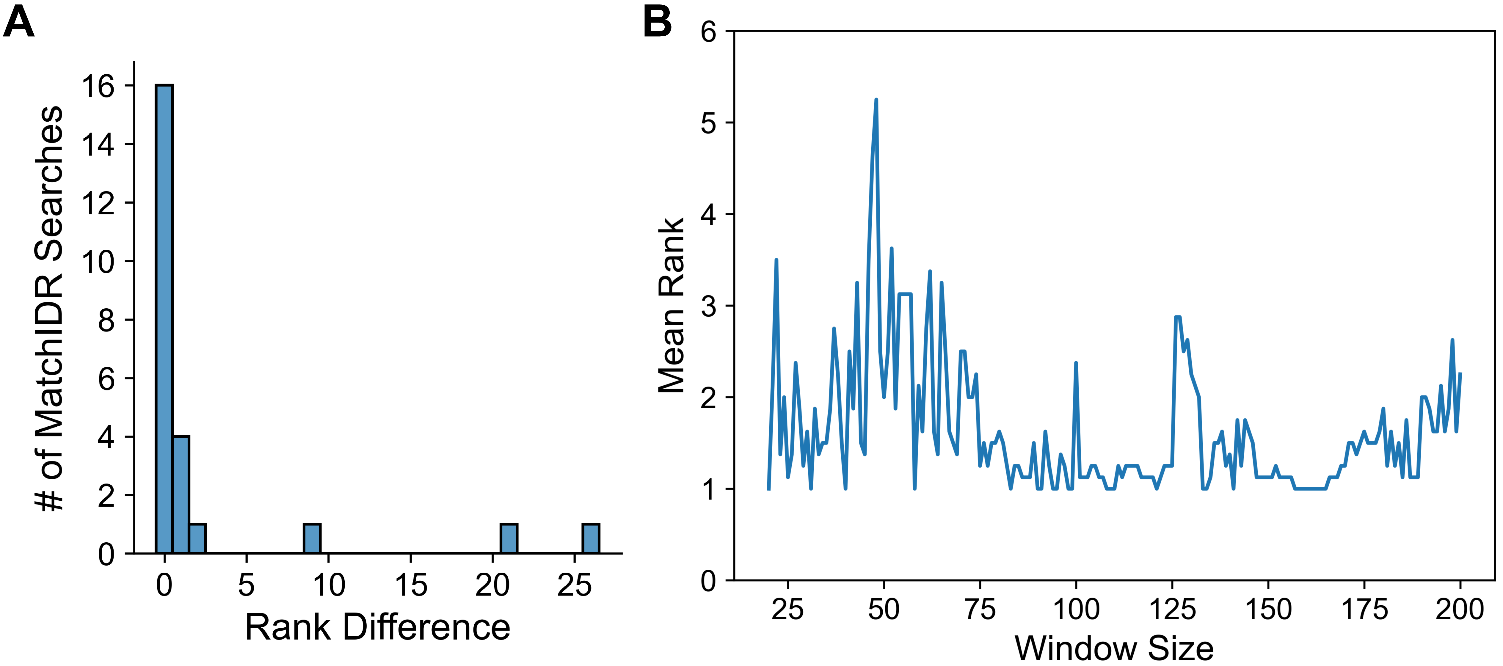


**FigS4. Dependence of MatchIDR ranking on distance metric.** (A) Bar plot showing the difference in ranking between the top MatchIDR hits using Manhattan distance and their rankings according to Euclidean distance for the 27 PrLDs experimentally tested in this study. The ranks of top hits from each original MatchIDR search were subtracted from the ranks of those same proteins in the corresponding MatchIDR results using the Euclidean distance (“Rank Difference”). (B) Mean rank of the best compositional match (Manhattan distance) among the MatchIDR results using the Euclidean distance, plotted as a function of window size.

Second, the MatchIDR searches of the yeast proteome using the eight PrLD query sequences for all window sizes from 20 to 200 (as described above) were repeated using the Euclidean distance metric. For each of the window sizes, the highest-ranking protein was collected from each query PrLD search, and the rank of this hit protein was determined among the corresponding MatchIDR search using the Euclidean distance. For each window size, the mean rank of hits was calculated across the eight query PrLDs (Fig S4B). The mean rank of top hits from the Manhattan distance among results from the Euclidean distance tend to be more volatile for smaller window sizes. However, the mean rank of the top Manhattan distance hits among the Euclidean distance results was almost always <5 (all but one of the tested window sizes), and 93.4% of the tested window sizes resulted in a top Manhattan distance hits having a mean rank within the top 3 Euclidean distance hits.

Fig S5

**
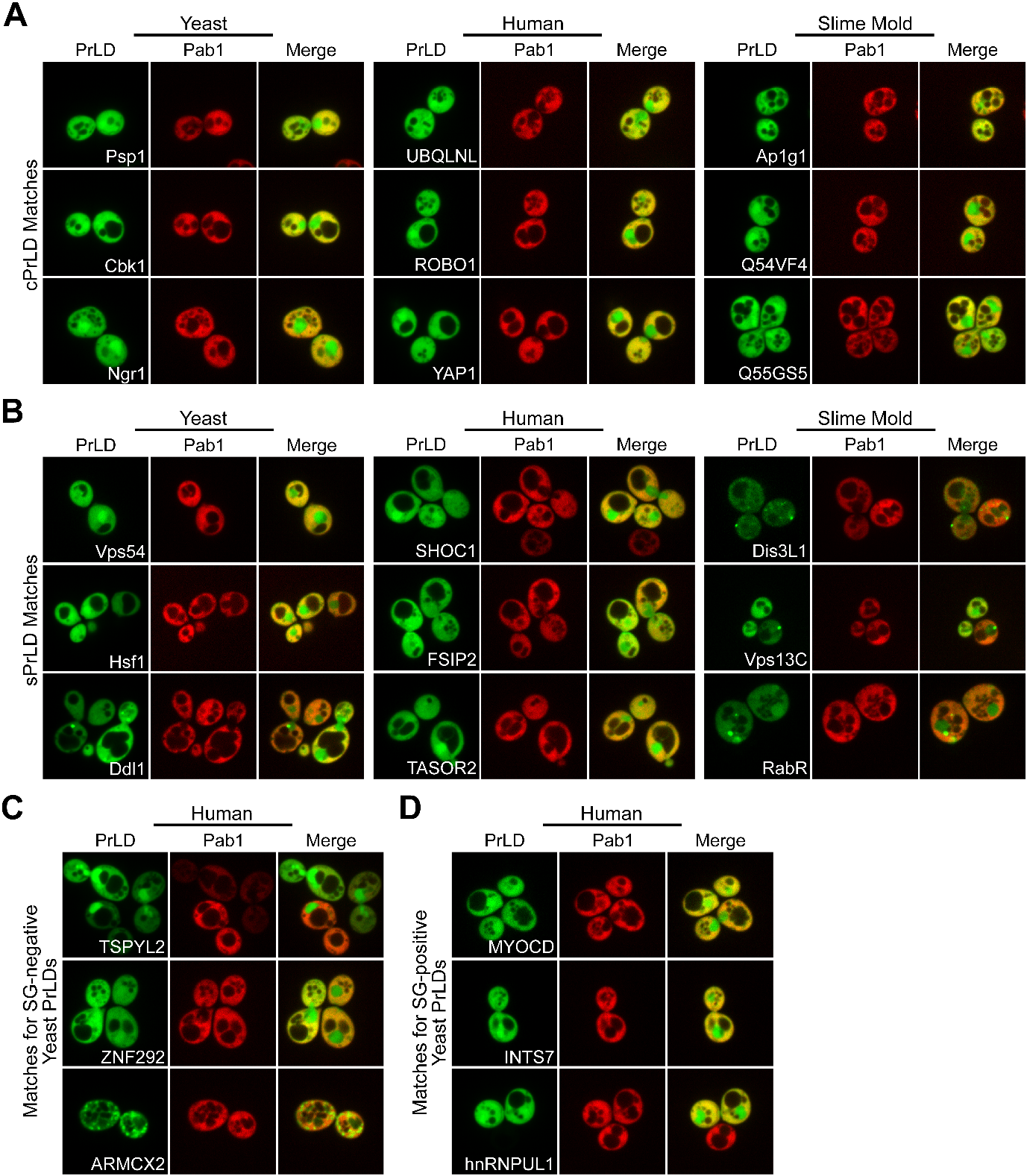
**

**Fig S5. Representative images of yeast expressing MatchIDR-identified PrLDs at the standard 30^o^C growth temperature.** Most PrLDs exhibited diffuse cytoplasmic localization at 30^o^C. For PrLDs with a small but reproducible subset of cells forming foci at 30^o^C (Ddl1, Dis3L1, Vps13C, RabR, and ARMCX2), representative images were selected to show at least one cell with a focus but do not reflect the frequency of foci-forming cells among the population.

Fig S6

**
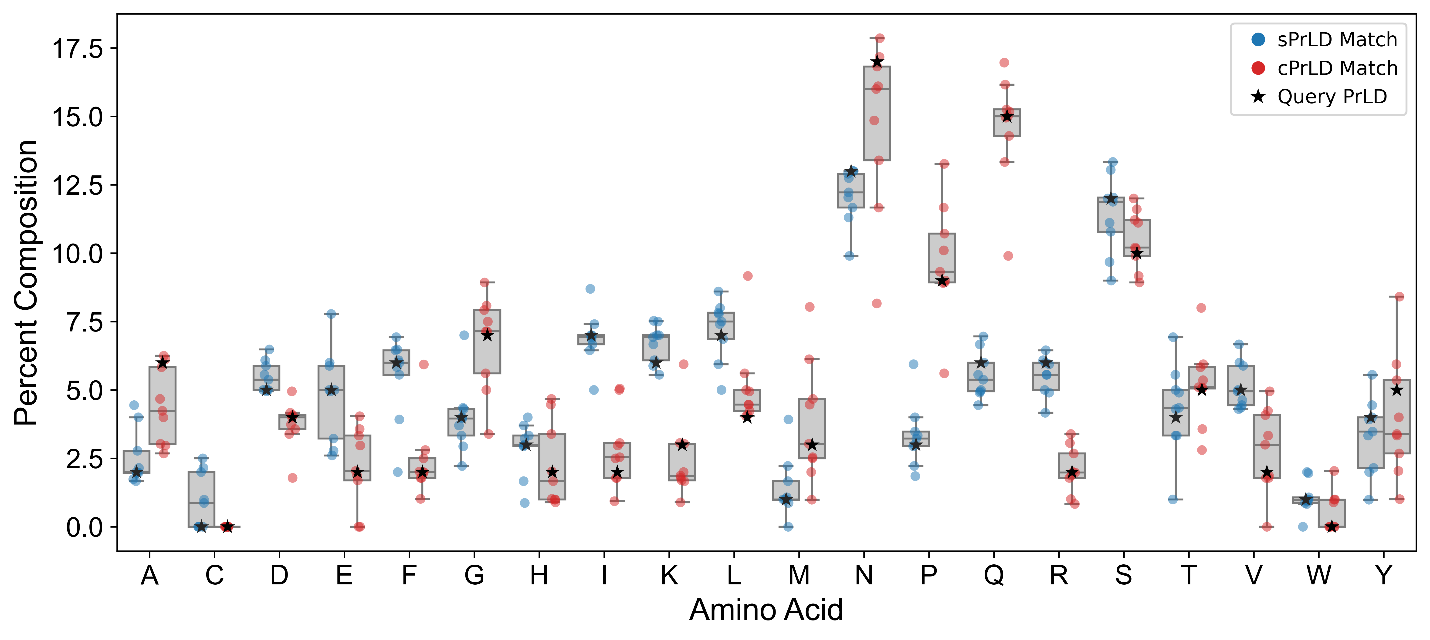
**

**Fig S6. Amino acid composition of sPrLD matches and cPrLD matches.** Percent composition for each amino acid was calculated for MatchIDR matches from yeast, humans, and slime mold and grouped based on the original query protein (sPrLD matches in blue, cPrLD matches in red). For comparison, the composition values for sPrLD and cPrLD are represented as black stars.

Fig S7


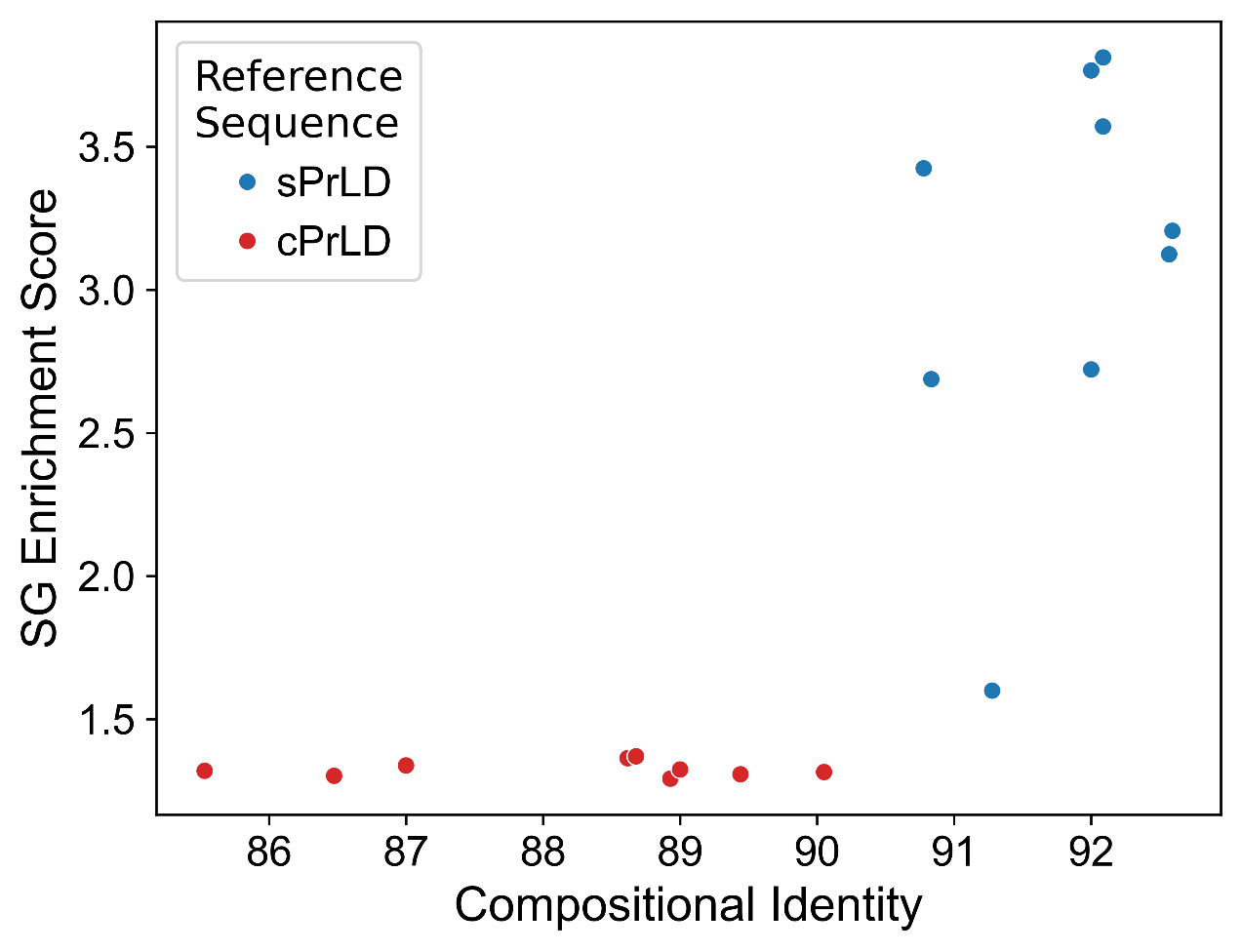


**Fig S7. SG enrichment score versus compositional identity for sPrLD and cPrLD matches.** MatchIDR matches for sPrLD (blue) and cPrLD (red) from yeast, humans, and slime mold are plotted with respect to mean SG enrichment score and compositional identity relative to their corresponding reference sequences.

Fig S8

**
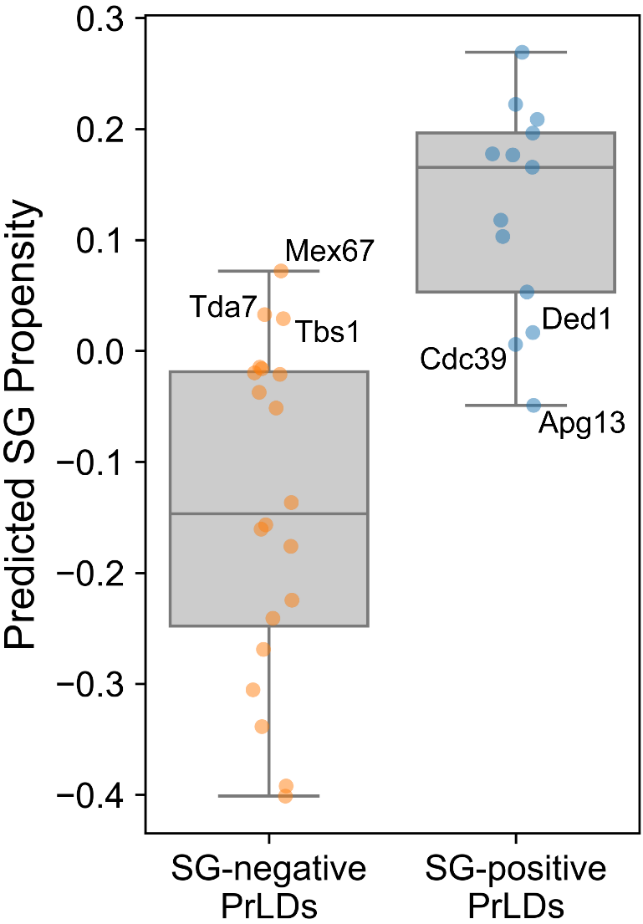
**

**Fig S8. SG-propensity scores for native yeast PrLDs with previously characterized SG-localization activity.** Predicted SG propensities for a set of native yeast PrLDs with SG enrichment (“SG-positive PrLDs”) or no detectable SG enrichment (“SG-negative PrLDs”) were reported in our prior study (Boncella *et al.*, 2020). The contribution of each amino acid to SG localization was estimated based on their enrichment or depletion in the SG-positive PrLD sequences relative to the SG-negative PrLD sequences. Predicted SG-propensity scores are the average estimated contribution of each residue across the PrLD sequence. The six native yeast PrLDs used as query sequences in MatchIDR searches (labeled proteins; this study) represent the most extreme inconsistencies between predicted SG propensity and observed SG-localization activity.

Fig S9


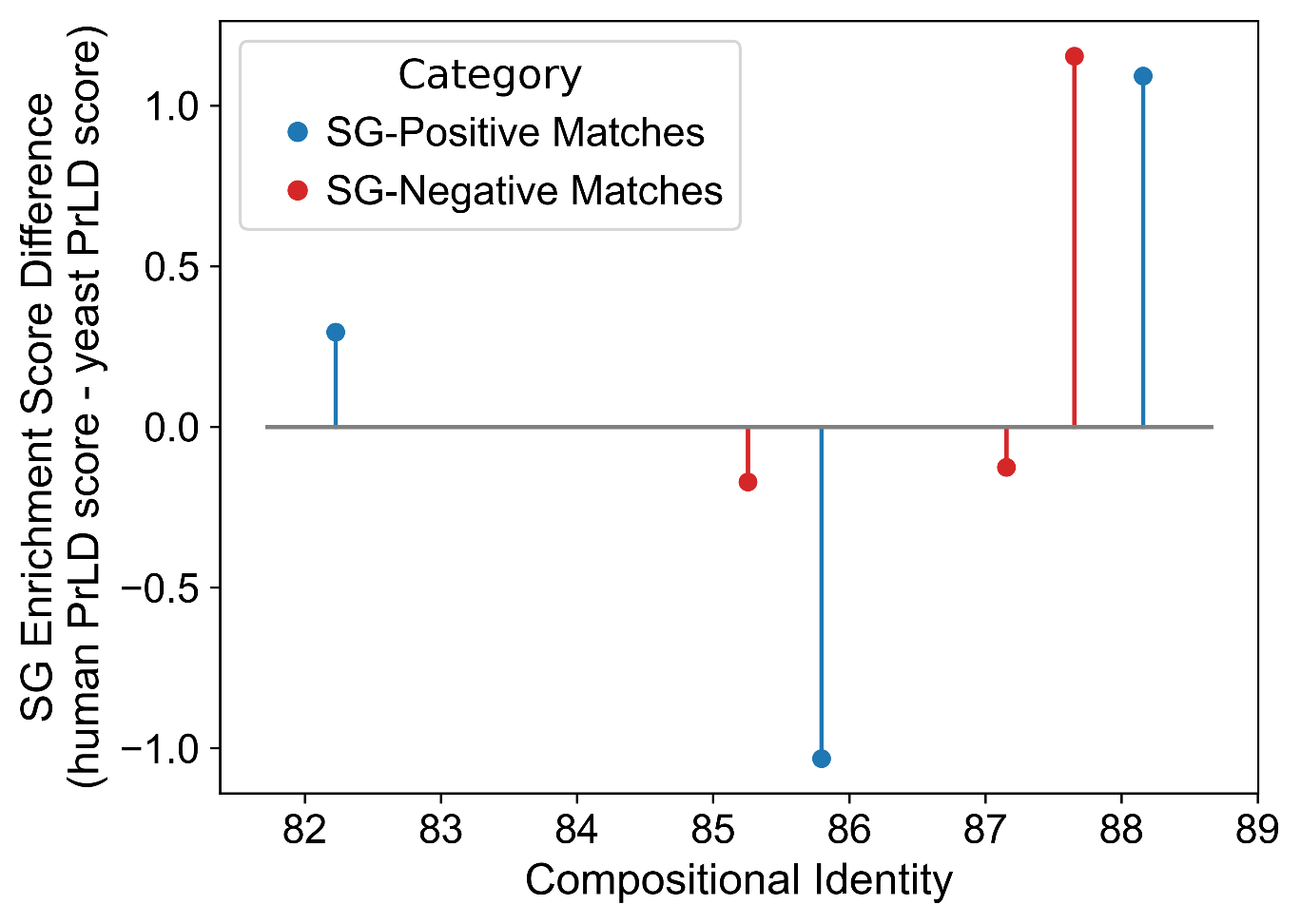


**Fig S9. SG enrichment score versus compositional identity for human PrLDs identified as top matches using native yeast PrLD query sequences.** Each human MatchIDR match was compared to its corresponding yeast PrLD query sequence with respect to both SG enrichment score and compositional identity. Values on the *y*-axis represent the difference in mean SG enrichment score for the human PrLD and the mean SG enrichment score for its yeast PrLD counterpart.

Fig S10


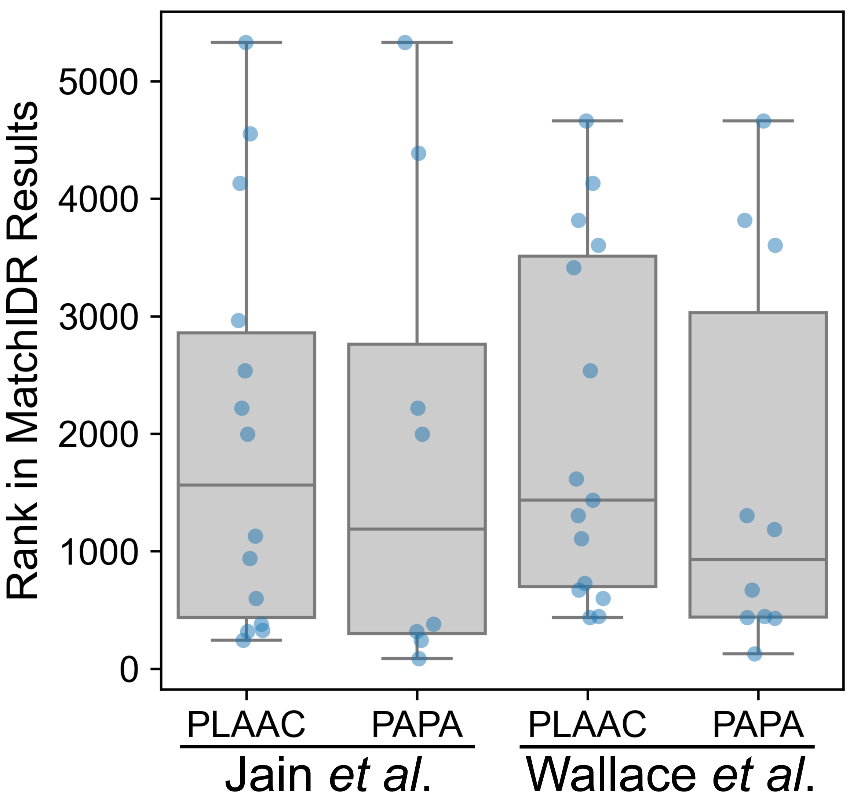


**Fig S10. Ranking of yeast SG proteins containing predicted PrLDs among MatchIDR results for the sPrLD query protein.** Yeast SG proteins from two different SG datasets (Wallace *et al.*, 2015; Jain *et al.*, 2016) were analyzed using two different prion prediction algorithms, PAPA and PLAAC (Toombs *et al.*, 2010; Lancaster *et al.*, 2014). Of the SG proteins with a predicted PrLD, the rank of each protein was determined among the MatchIDR results generated by using the sPrLD query protein, a window size range of 90-120, and the Manhattan distance metric.

Fig S11


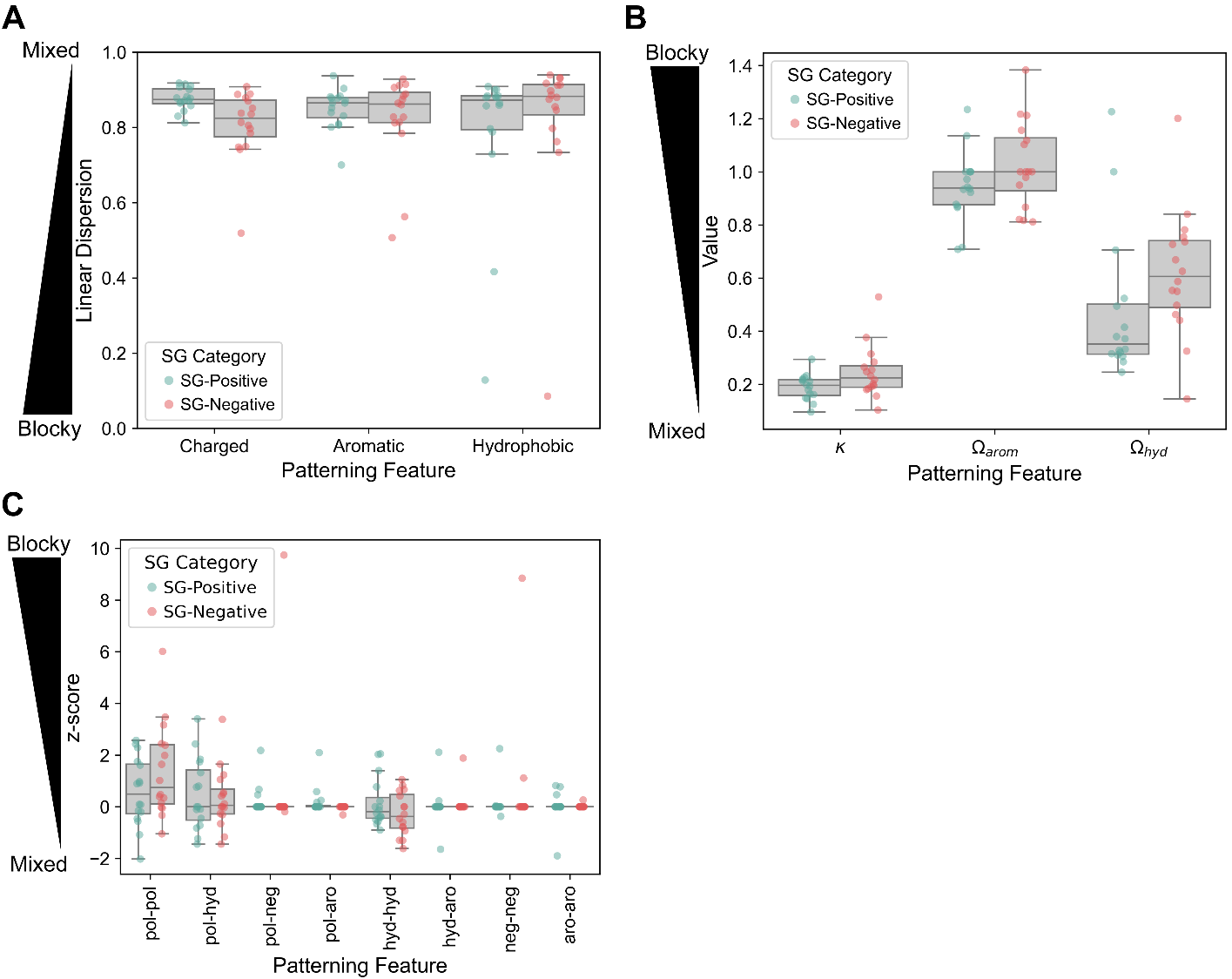


**Fig S11. Measures of sequence patterning among SG-localizing and non-SG-localizing PrLDs.** (A) Linear dispersion of charged (D/E/H/K/R), aromatic (F/W/Y), and hydrophobic (I/L/M/V) amino acids according to the linear dispersion method used in LCD-Composer (Cascarina *et al.*, 2021). (B) Patterning features κ (distribution of D/E relative to K/R), Ω*_arom_* (distribution of F/W/Y relative to all other residues), and Ω*_hyd_* (distribution of I/L/V relative to all other residues) according to localCIDER (Holehouse *et al.*, 2017). Despite normalization, some values can exceed 1.0 when the residues involved in the patterning feature constitute a small fraction of the sequence (Ginell and Holehouse, 2020; Cohan *et al.*, 2022). (C) Patterning of various groups of residues according to NARDINI+ (Cohan *et al.*, 2022; Ruff *et al.*, 2026). Residue groups are pol (S/T/N/Q/C/H), hyd (I/L/M/V), pos (K/R), neg (D/E), aro (F/W/Y). Patterning features with the same label repeated (e.g., pol-pol) represent patterning of those residues relative to all other types of residues not in that group. NARDINI+ assigns an arbitrary *z*-score of 0 to a sequence if the total fraction of residues belonging to either group in the patterning feature constitute <10% of the sequence composition. While all 36 patterning features were evaluated for the SG proteins, only those with at least one non-zero value for the SG-positive and SG-negative groups are shown.

Supplementary Table Legends

**Table S1. MatchIDR search results from *Saccharomyces cerevisiae* for the sPrLD and cPrLD query proteins.** See “MatchIDR Output” section on page 2 for a description of data fields.

**Table S2. *p*-values for SG enrichment scores.** Two-tailed Welch’s *t*-tests were performed to determine if the degree of SG localization differed significantly from a protein with no detectable SG enrichment (cPrLD2). For each comparison, the SG enrichment scores of that protein are compared to the SG enrichment scores for cPrLD2 from Fig 2.

**Table S3. MatchIDR search results from *Homo sapiens* for the sPrLD and cPrLD query proteins.** See “MatchIDR Output” section on page 2 for a description of data fields.

**Table S4. MatchIDR search results from *Dictyostelium discoideum* for the sPrLD and cPrLD query proteins.** See “MatchIDR Output” section on page 2 for a description of data fields.

**Table S5. MatchIDR search results from *Homo sapiens* for the native yeast PrLD query proteins.** See “MatchIDR Output” section on page 2 for a description of data fields.

References

Boncella, AE, Shattuck, JE, Cascarina, SM, Paul, KR, Baer, MH, Fomicheva, A, Lamb, AK, and Ross, ED (2020). Composition-based prediction and rational manipulation of prion-like domain recruitment to stress granules. Proc Natl Acad Sci U S A 117, 5826–5835.

Cascarina, SM, King, DC, Osborne Nishimura, E, and Ross, ED (2021). LCD-Composer: an intuitive, composition-centric method enabling the identification and detailed functional mapping of low-complexity domains. NAR Genom Bioinform 3, lqab048.

Cascarina, SM, and Ross, ED (2025). Protein activities driven by amino acid composition. Journal of Biological Chemistry, 110640.

Cohan, MC, Shinn, MK, Lalmansingh, JM, and Pappu, R V. (2022). Uncovering Non-random Binary Patterns Within Sequences of Intrinsically Disordered Proteins. J Mol Biol 434, 167373.

Ginell, GM, and Holehouse, AS (2020). Analyzing the Sequences of Intrinsically Disordered Regions with CIDER and localCIDER. Methods in Molecular Biology 2141, 103–126.

Holehouse, AS, Das, RK, Ahad, JN, Richardson, MOG, and Pappu, R V. (2017). CIDER: Resources to Analyze Sequence-Ensemble Relationships of Intrinsically Disordered Proteins. Biophys J 112, 16–21.

Jain, S, Wheeler, JR, Walters, RW, Agrawal, A, Barsic, A, and Parker, R (2016). ATPase-Modulated Stress Granules Contain a Diverse Proteome and Substructure. Cell 164, 487–498.

Lancaster, AK, Nutter-Upham, A, Lindquist, S, and King, OD (2014). PLAAC: a web and command-line application to identify proteins with prion-like amino acid composition. Bioinformatics 30, 2501–2502.

Ruff, KM, King, MR, Ying, AW, Liu, V, Pant, A, Lieberman, WE, Shinn, MK, Su, X, Kadoch, C, and Pappu, R V. (2026). Molecular grammars of predicted intrinsically disordered regions that span the human proteome. Cell 189, 323-342.e17.

Toombs, JA, McCarty, BR, and Ross, ED (2010). Compositional Determinants of Prion Formation in Yeast. Mol Cell Biol 30, 319–332.

Wallace, EWJ, Kear-Scott, JL, Pilipenko, E V., Schwartz, MH, Laskowski, PR, Rojek, AE, Katanski, CD, Riback, JA, Dion, MF, Franks, AM, *et al.* (2015). Reversible, Specific, Active Aggregates of Endogenous Proteins Assemble upon Heat Stress. Cell 162, 1286–1298.
